# Supplementary material for: WNK1 signalling regulates amino acid transport and mTORC1 activity to sustain acute myeloid leukaemia growth
Source: Nat Commun. 2025 May 27;16:4920. doi: 10.1038/s41467-025-59969-8 (PMC12116911; doi:10.1038/s41467-025-59969-8)

Supplementary Fig. 1

a

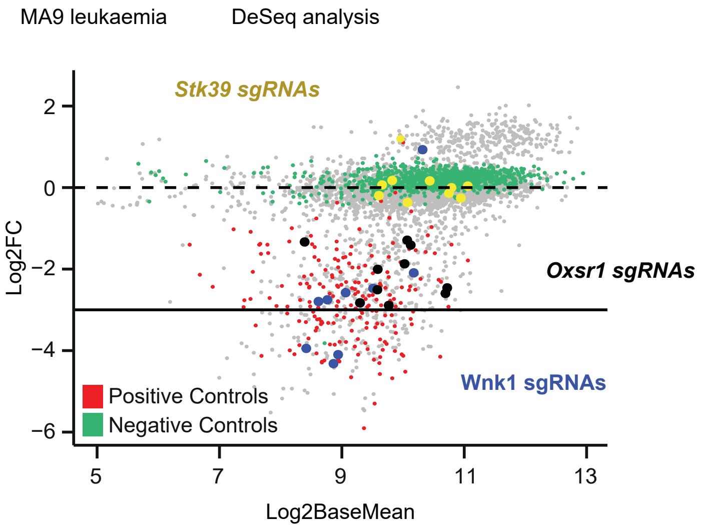

b

Screen data
• 6237 sgRNA library targeting 525 mouse kinases (3-10 sgRNAs/gene; 1000 negative controls and 100 positive controls)
• 129 sgRNA identified, targeting 37 different kinases, incl. CDK1,CDK9,PLK1,BUB1,ATR,CHK1,JAK2, and MTOR.

c

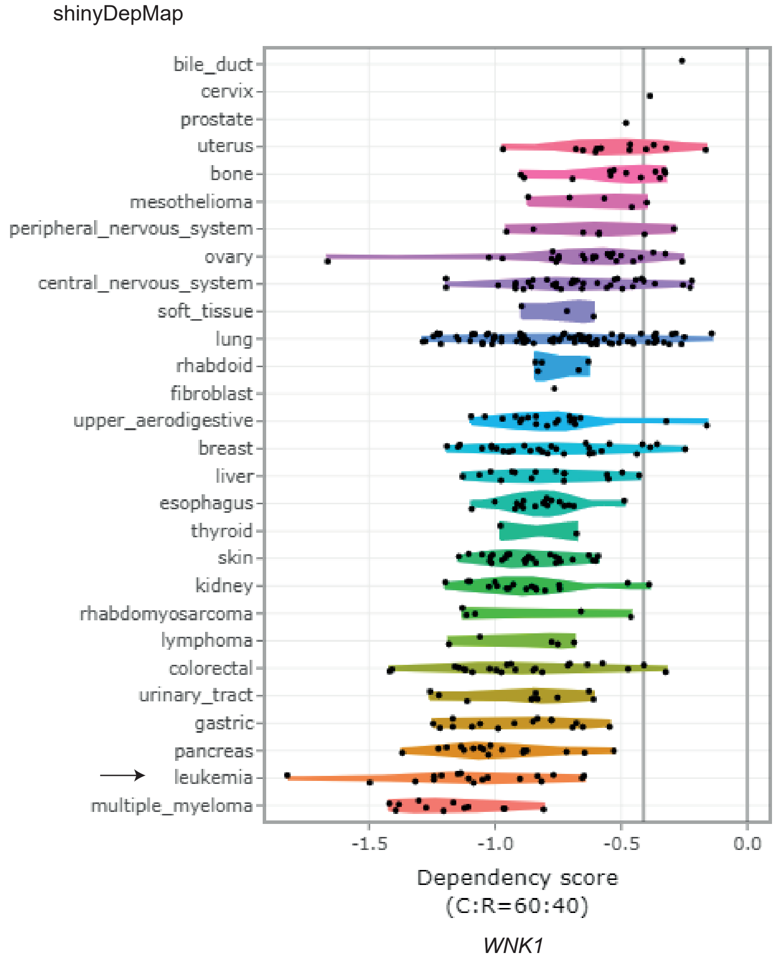

Supplementary Fig.1. Identification of WNK1 as an essential kinase for AML.

(**a**) DeSeq2 analysis of the screening results. sgRNAs for positive control genes are shown in red, sgRNAs for negative control genes in green, sgRNAs for *Wnk1* in purple, sgRNAs for *Oxsr1* in black, and sgRNAs for *Stk39* in yellow. (**b**) An overview of the composition of the sgRNA library targeting the mouse kinome and representative kinases that were identified are shown. (**c**) The dependency scores of *WNK1* are grouped by lineages using shinyDepMap (https://labsyspharm.shinyapps.io/depmap).

Supplementary Fig. 2

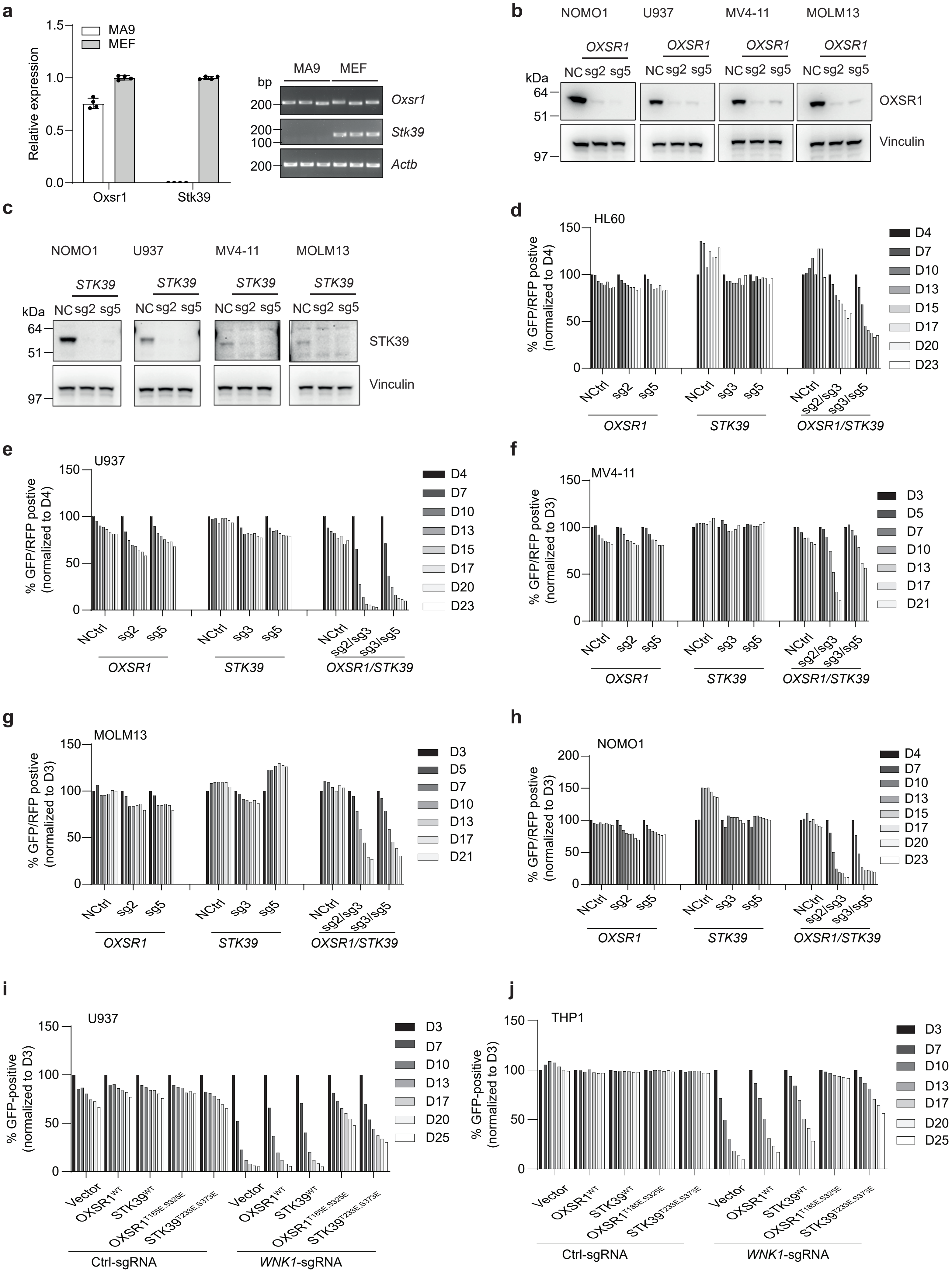

Supplementary Fig.2. OXSR1/STK39 mediates the requirement of WNK1 for AML.

(**a**) Left panel: qPCR of *Oxsr1* and *Stk39* in MA9 and MEF cells. Right panel: qPCR products were separated by electrophoresis on a 2% agarose gel. The data shown in the agarose gel represent three biological replicates (n=3). (**b**) Immunoblot of OXSR1 in Cas9-expressing human leukaemia cells that express either a non-targeting sgRNA or two independent sgRNAs targeting *Oxsr1*. The blots for each AML cell line are from one representative experiment (n=1). The samples were derived from the same experiment, and the same gel for OXSR1 and Vinculin. Vinculin served as a loading control. (**c**) Immunoblot of STK39 in Cas9-expressing human leukaemia cells that express either a non-targeting sgRNA or two independent sgRNAs targeting *STK39*. The blots for each AML cell line are from one representative experiment (n=1). The samples were derived from the same experiment, and the same gel for STK39 and Vinculin. Vinculin served as a loading control. (**d-h**) Drop-out growth competition assays, showing the relative percentage over time of the indicated sgRNA-positive (GFP for *OXSR1* sgRNA and RFP for *STK39* sgRNA) Cas9-expressing human AML cells. An sgRNA against an essential gene (*RPS19*) was used as a positive control, and a non-targeting sgRNA (NCtrl) was used as a negative control. Data shown for each cell line are from one representative experiment (n=1). (**i-j**) Drop-out growth competition assays, depicting the relative percentage over time of the WNK1 sgRNA-positive (GFP positive) Cas9-expressing human leukaemia cells that express $OXSR1^{WT}$, $STK39^{WT}$, $OXSR1^{T185E, S325E}$ or $STK39^{T233E, S373E}$. Data shown for each cell line are from one representative experiment (n=1).

Supplementary Fig. 3

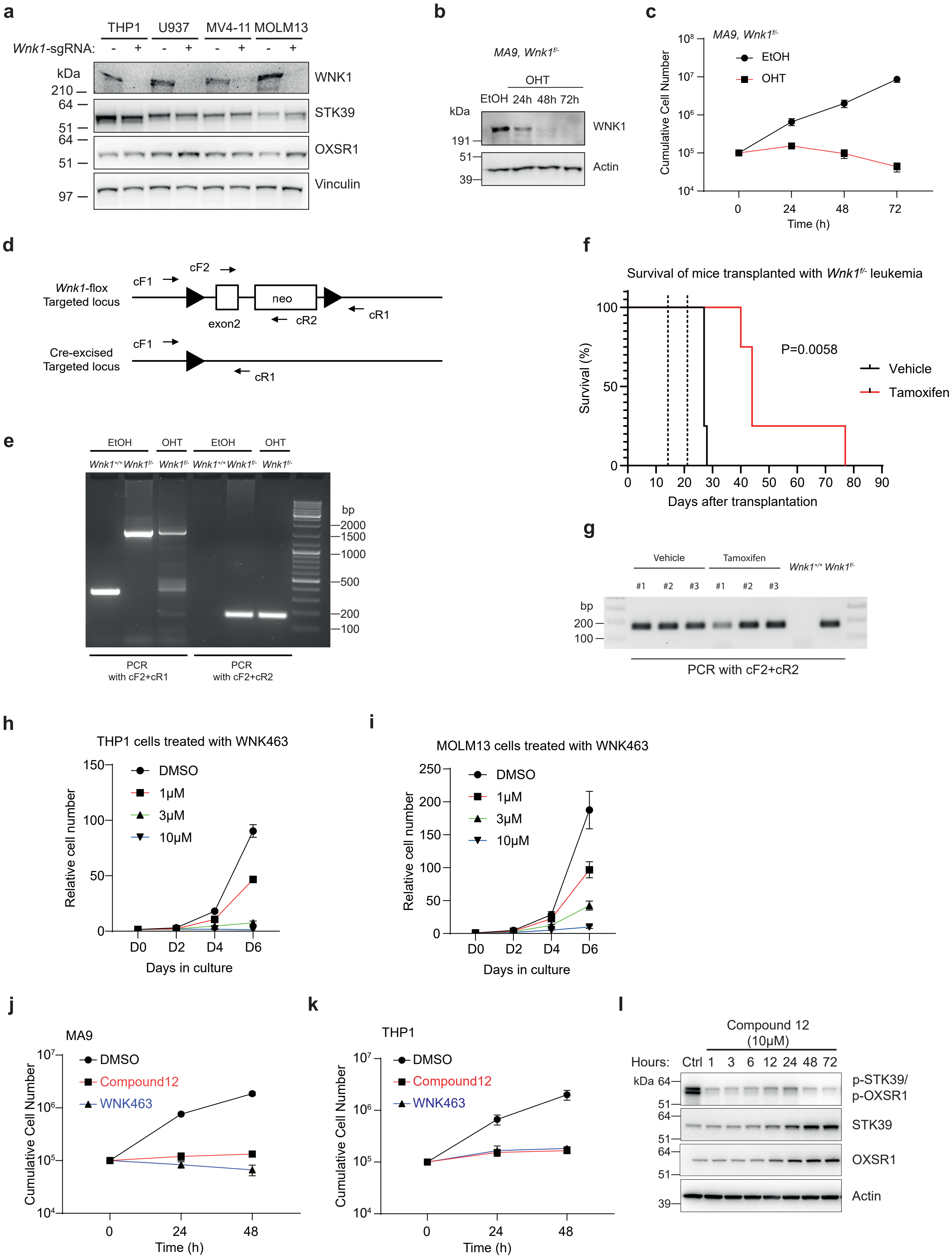

Supplementary Fig.3. WNK1 depletion impairs AML proliferation *in vitro*.

(**a**) Immunoblot analysis of OXSR1 and STK39 following WNK1 knockdown in human leukaemia cells. The blots are from one representative experiment (n=1). The samples were derived from the same experiment, but different gels for WNK1 and OXSR1, another for STK39 and Vinculin were processed in parallel. Vinculin served as a loading control. (**b**) Immunoblot of WNK1 in $Wnk1^{fl/-}$ MA9 leukaemia cells in the presence of OHT (500 nM) for the indicated time. The blots are representative of two independent experiments (n=2). The samples were derived from the same experiment, and the same gels for WNK1 and Actin. Actin served as a loading control. (**c**) Growth curve of $Wnk1^{fl/-}$ leukaemia cells treated with 500 nM OHT for the indicated time. Ethanol (EtOH) was used as a control. Data are presented as mean ± SD of technical replicates and are representative of at least three independent experiments (n>3). (**d**) Schematic for the floxed *Wnk1* locus. (**e**) Genotyping of OHT-resistant MA9 $Wnk1^{fl/-}$ leukaemia cells with the indicated primers. The gel is from one representative experiment (n=1). (**f**) Replicate of experiment shown in Figure 3f. Kaplan–Meier survival curves of recipient mice transplanted with the indicated leukaemia cells. Statistical significance was calculated using a log-rank test. (**g**) Representative genotyping of bone marrow from tamoxifen-treated recipient mice transplanted with $Wnk1^{fl/-}$ MA9 leukaemia cells at the endpoint shown in (**f**). $Wnk1^{+/+}$ and parental $Wnk1^{fl/-}$ cells were used as controls for PCR analysis. The gel is from one representative experiment with three biological replicates (n=3 for vehicle, and n=3 for tamoxifen). **(h-i)** Growth curve of human THP1 (**h**) and human MOLM13 (**i**) leukaemia cells treated with WNK463 at the indicated concentrations. Data are presented as mean ± SD of three independent experiments (n=3). **(j-k)** Growth curves of MA9 (**j**) and THP1 (**k**) leukaemia cells treated with either WNK463 or Compound 12 both at 10 µM. Data are presented from one representative experiment (n=1) **(l)** Western blot analysis of phospho-STK39 (S373) and phospho-OXSR1 (S325), as well as total OXSR1 and STK39, in human THP-1 lysates treated with 10 µM Compound 12 at the indicated time points. The samples were derived from the same experiment, but different gels for p-STK39/p-OXSR1, another for OXSR1, another for STK39, and another for Actin were processed in parallel. Actin was used as a loading control. The blots are representative of two independent experiments (n=2).

Supplementary Fig. 4

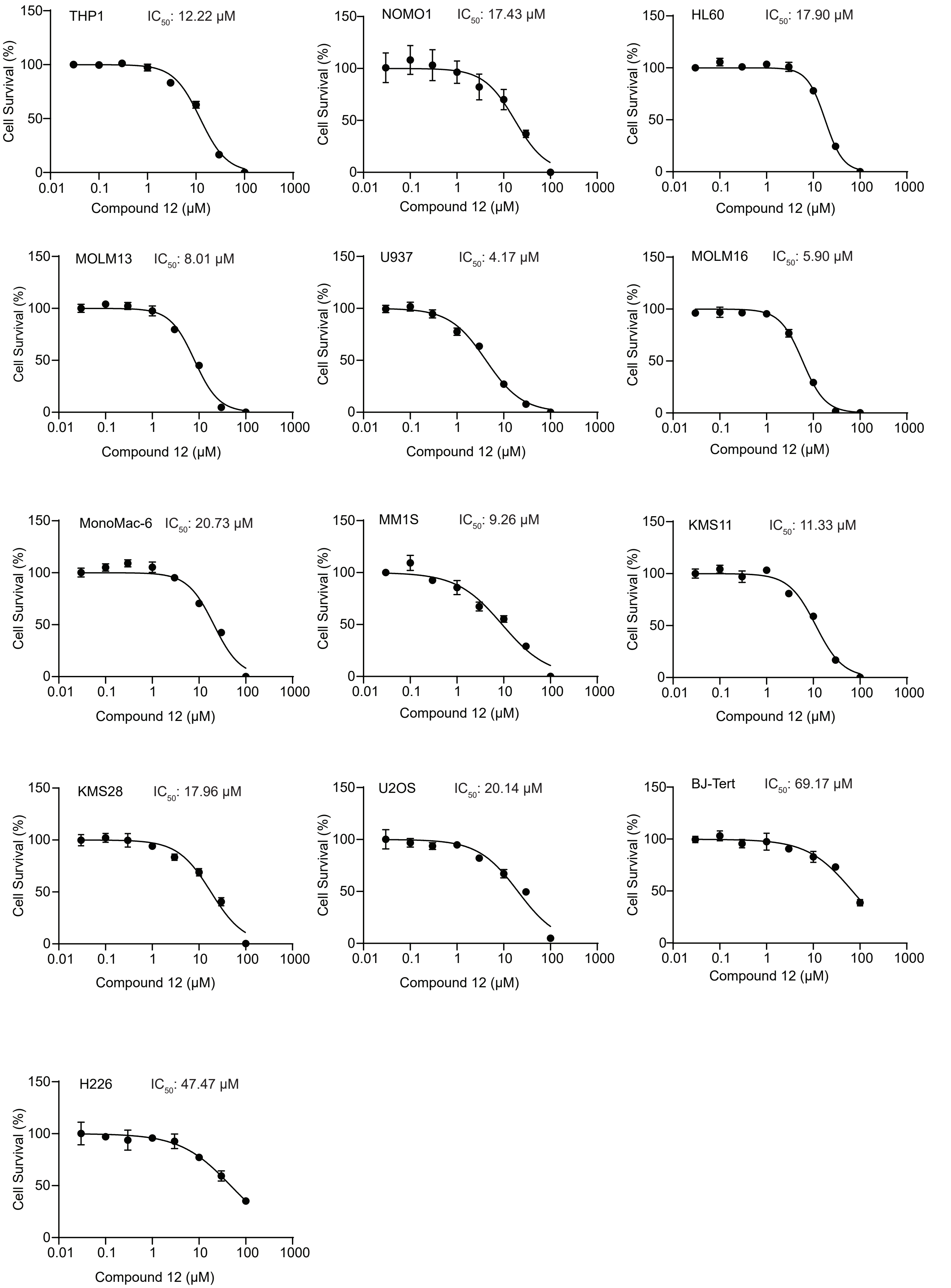

Supplementary Fig.4. Compound 12 displays anti-leukaemia activities in diverse human AML cell lines. Dose response of cell viability of the indicated cell lines to treatment with Compound 12. Data are presented as mean ± SD of three biological replicates (n=3).

Supplementary Fig. 5

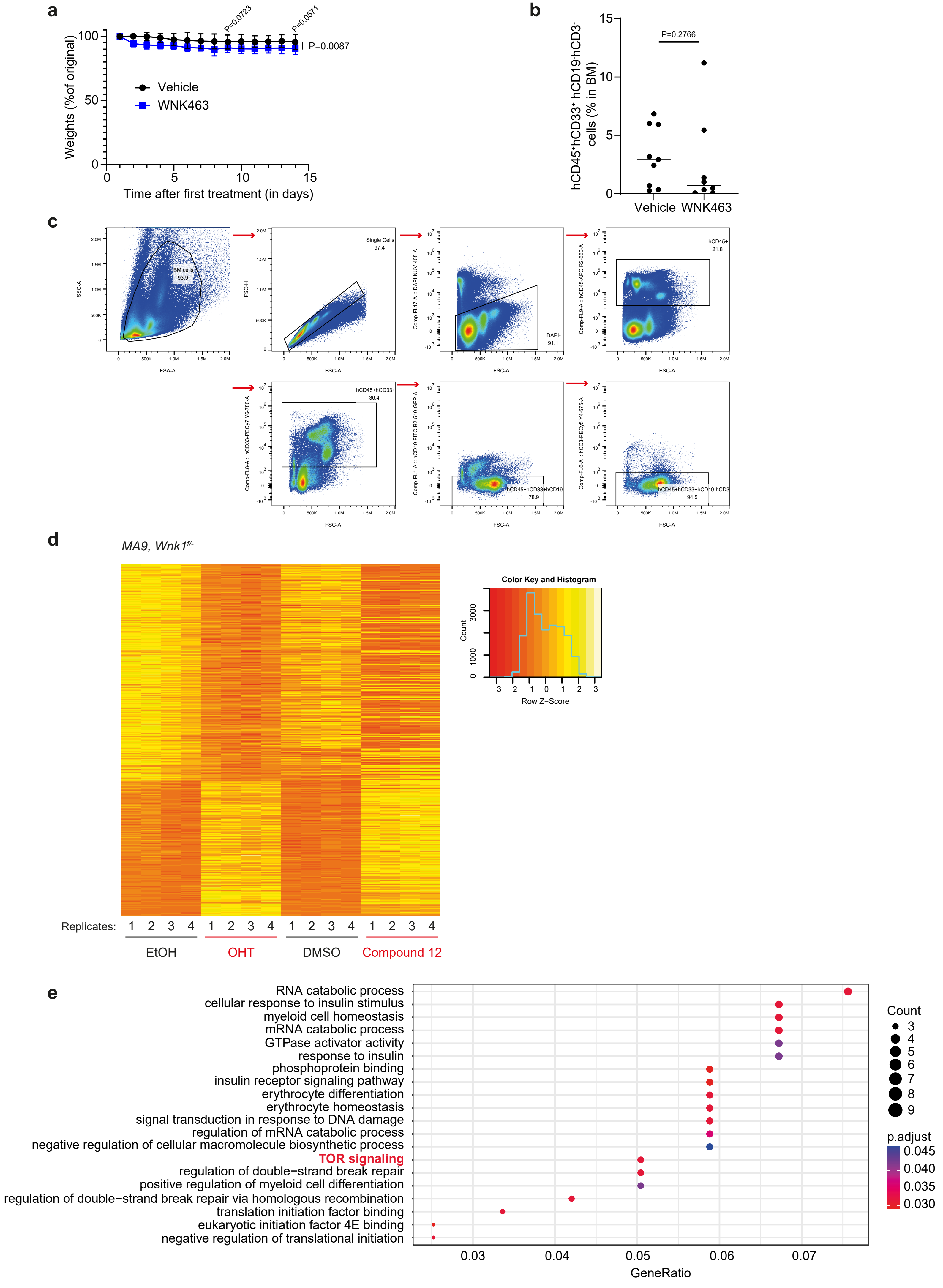

Supplementary Fig.5. Identification of WNK1-OXSR1/STK39 pathway dependent phosphorylation sites in the proteome.

(**a**) Body weights of mice treated with either vehicle or WNK463 (1.5 mg/kg, administered orally, twice daily) for 14 consecutive days. (n = 9 for Vehicle, n = 8 for WNK463-treated). The weights are presented as a percentage of their original weights before treatment. A two-way ANOVA was used to compare the Vehicle and WNK463-treated groups, and a post-hoc test with Tukey's test was used to compare the two groups at each time point. (**b**) Percentage of human viable AML cells expressing CD45 and CD33 and not expressing CD19 and CD3 ($hCD45^{+}hCD33^{+}hCD19^{-}hCD3^{-}$) in bone marrow of mice bearing primary human AML cells that were treated with vehicle or WNK463. The percentage of cells was determined by FACS analysis after treatment with either vehicle or WNK463 (1.5 mg/kg, administered orally, twice daily) for 14 consecutive days. (n = 9 for Vehicle, n = 8 for WNK463-treated). Statistical significance was determined using the Mann-Whitney U test (two-tailed, unpaired). ns, not significant. (**c**) Flow cytometry plots indicating the gating strategy to identify live hCD45+hCD33+hCD19-hCD3- cells. (**d**) Heat map of significantly changing phosphosites in *Wnk1*$^{fl/-}$ MA9 leukaemia cells treated with 500 nM OHT for 48 h or 30 µM Compound 12 for 3h. (**e**) Gene ontology enrichment analysis of the common significantly regulated phosphosites (FDR<0.05) between WNK1 inhibitor treatment and WNK1 knockout conditions. The analysis focuses on 120 proteins having the phosphosites with the highest overall changes (the top 20% highest changing phosphosites).

Supplementary Fig. 6

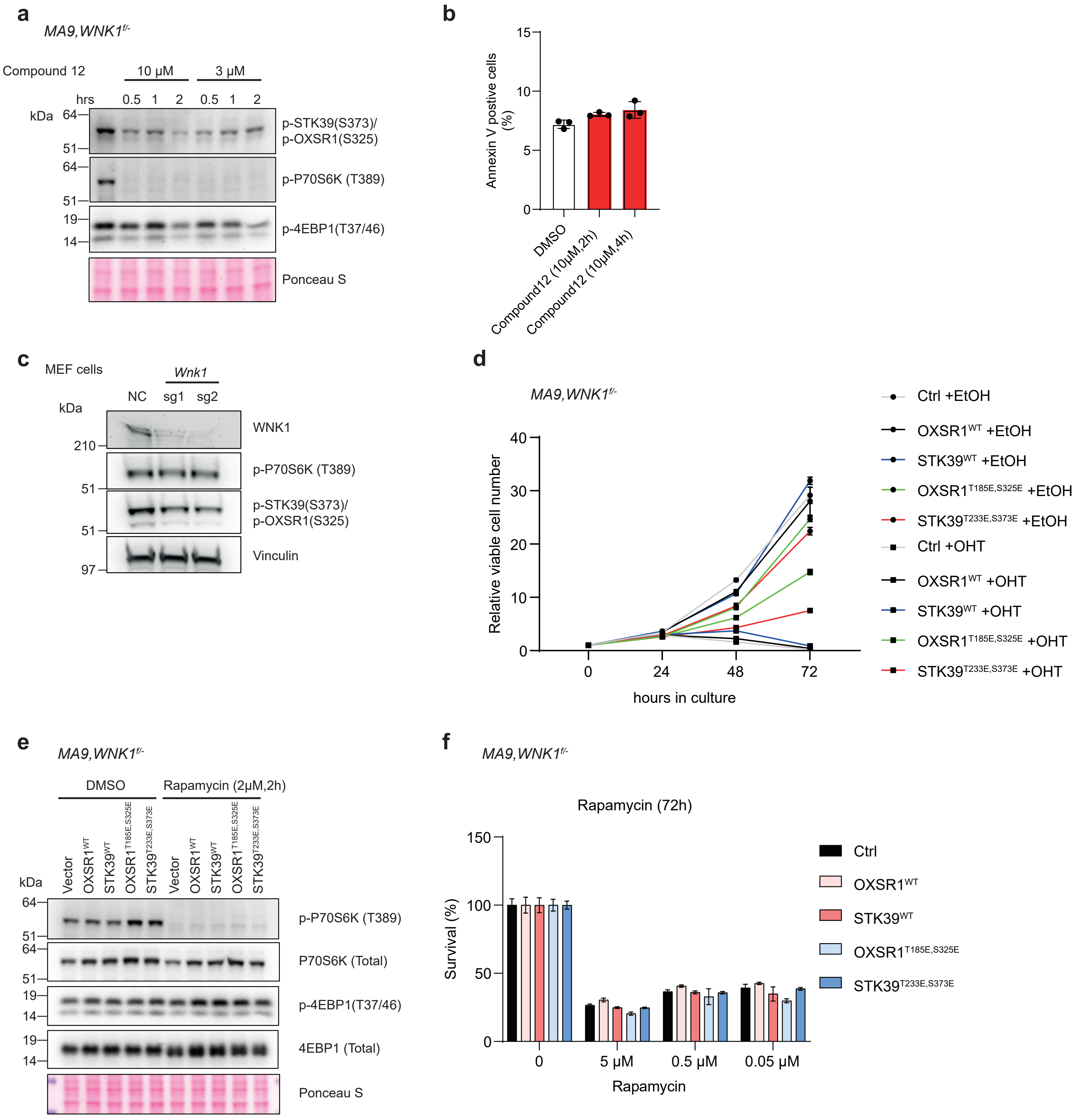

Supplementary Fig.6. WNK1-OXSR1/STK39 acts upstream of mTORC1 signalling.

(**a**) Immunoblot analysis of the indicated proteins in *Wnk1*$^{fl/-}$ MA9 leukaemia cells in the presence of Compound 12 at the indicated concentration and time points. The blots are representative of two independent experiments (n=2). The samples were derived from the same experiment, but different gels for p-STK39(S373)/p-OXSR1(S325), and another for p-P70S6K (T389) and p-4EBP1(T37/46) were processed in parallel. Ponceau S staining served as a loading control. (**b**) Analysis of cell apoptosis measured by APC-conjugated Annexin V staining in MA9 leukaemia cells following treatment with 10 µM Compound 12 at the indicated time points. Data are presented as mean ± SD from three biological replicates (n=3). (**c**) Immunoblot analysis of the indicated proteins in *Cas9*-expressing MEF cells that express either a non-targeting sgRNA (NC) or two independent sgRNAs targeting *Wnk1.* The blots are from one representative experiment (n=1). The samples were derived from the same experiment, but different gels for WNK1 and p-STK39(S373)/p-OXSR1(S325), and another for p-P70S6K (T389) and Vinculin were processed in parallel. Vinculin served as a loading control. (**d**) Growth curve of *Wnk1*$^{fl/-}$ MA9 leukaemia cells ectopically expressing OXSR1^{WT}, STK39^{WT}, OXSR1$^{T185E, S325E}$ or STK39$^{T233E, S373E}$ in the presence of EtOH or 500 nM OHT. Data are presented as mean ± SD of three biological replicates (n=3). (**e**) Immunoblot analysis of the indicated proteins of *Wnk1*$^{fl/-}$ MA9 leukaemia cells ectopically expressing OXSR1^{WT}, STK39^{WT}, OXSR1$^{T185E, S325E}$ or STK39$^{T233E, S373E}$ in the presence of DMSO or 2 µM rapamycin. The blots are from one representative experiment (n=1). The samples were derived from the same experiment, but different gels for p-P70S6K (T389) and p-4EBP1(T37/46), and another for P70S6K (Total) and 4EBP1 (Total) were processed in parallel. Ponceau S staining served as a loading control. (**f**) Cell viability of *Wnk1*$^{fl/-}$ MA9 leukaemia cells ectopically expressing OXSR1^{WT}, STK39^{WT}, OXSR1$^{T185E, S325E}$ or STK39$^{T233E, S373E}$ after treatment with DMSO or rapamycin for 72 h at the indicated concentrations. Data are mean ± SD of three biological replicates (n=3).

Supplementary Fig. 7

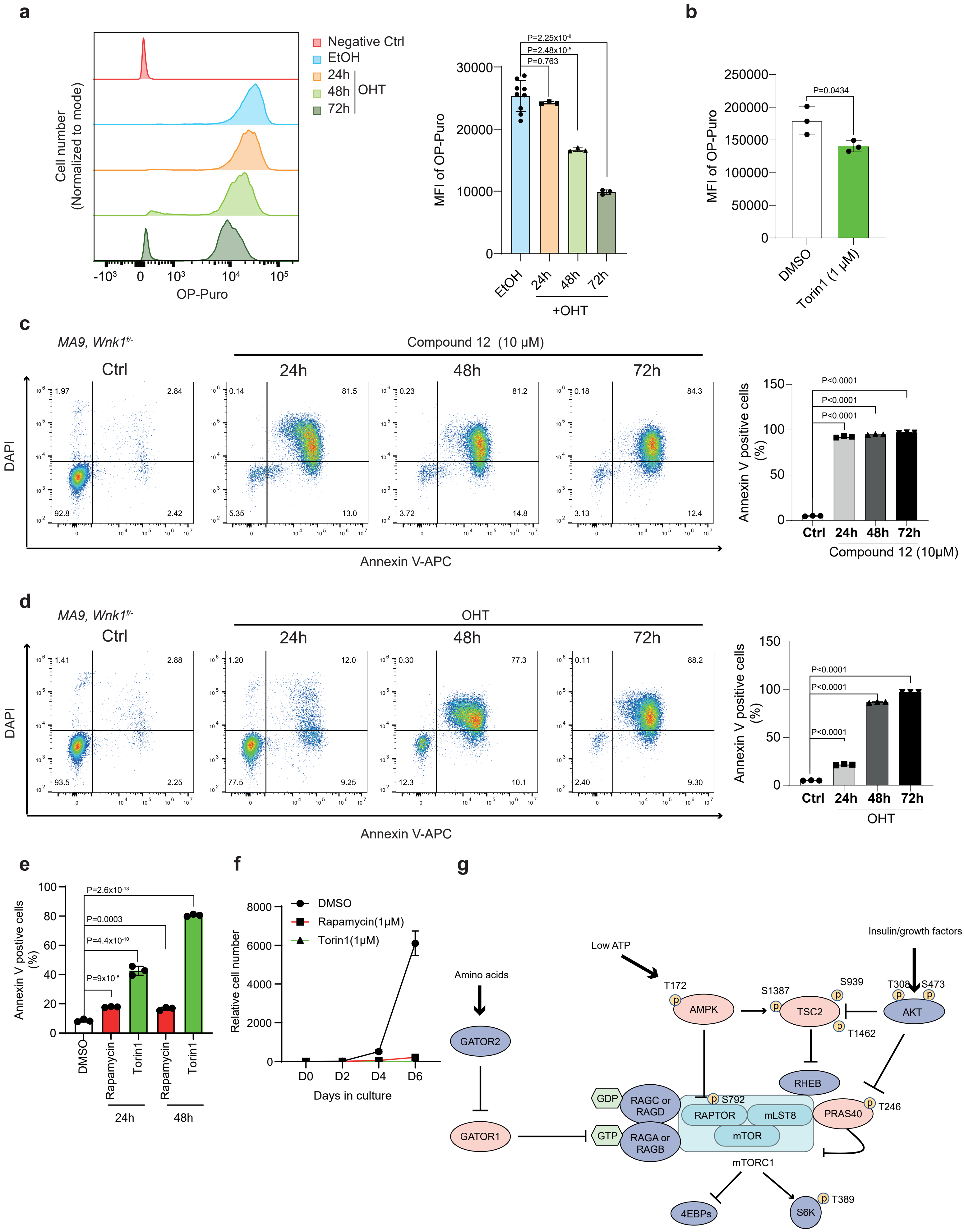

Supplementary Fig.7. Depletion/inhibition of WNK1 leads to a decrease of protein synthesis and an increase of apoptosis.

(**a-b**) Protein synthesis rates were measured by incorporation of OP-puro in *Wnk1*$^{fl/-}$ MA9 leukaemia cells after treatment with 500 nM OHT at the indicated time points or 1 µM mTOR kinase inhibitor Torin1 for 6 hours. Error bars represent mean ± SD from three biological replicates for OHT-treated time points (n=3) and nine biological replicates for EtOH-treated control (n=9). Statistical analysis was performed using one-way ANOVA followed by Dunnett's post-hoc test to compare each OHT-treated time point to the EtOH-treated control. Two-sided unpaired Student's t-test was used to compare the Torin1-treated group with the DMSO-treated control group.(**c-e**) Cell apoptosis analysis was measured by APC-conjugated Annexin V staining in *Wnk1*$^{fl/-}$ MA9 leukaemia cells after treatment with 10 µM Compound 12 (**c**) , 500 nM OHT (**d**) , 1 µM Rapamycin (**e**), or 1 µM Torin1 (**e**) at the indicated time points. Data are presented as mean ± SD of three biological replicates (n=3). Statistical analysis was performed using one-way ANOVA followed by Dunnett's post-hoc test to compare: each Compound12-treated time point to the untreated control(**c**); each OHT-treated time point to the EtOH-treated control(**d**); each Rapamycin- or Torin1-treated time point to the DMSO-treated control(**e**). (**f**) Growth curve of MA9 leukaemia cells *in vitro* treated with/without 1 µM Rapamycin or 1 µM Torin1. Data are presented as mean ± SD of three biological replicates (n=3). (**g**) Schematic depicting upstream regulators of mTORC1 signalling.

Supplementary Fig. 8

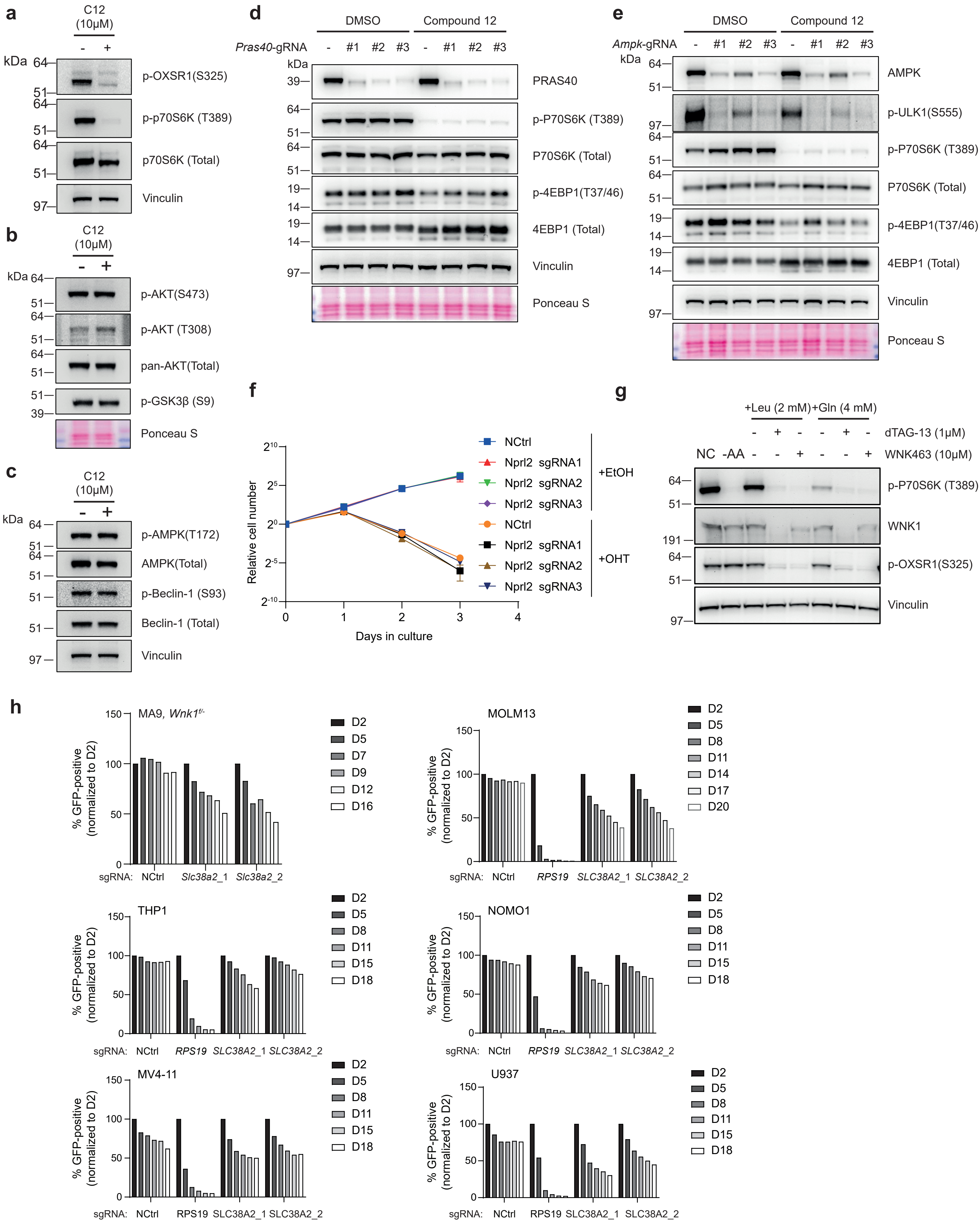

Supplementary Fig.8. WNK1 inhibition does not affect PI3K-AKT and AMPK signals.

(**a-c**) Immunoblot analysis of the indicated proteins in *Wnk1*$^{fl/-}$ MA9 leukaemia cells in the presence of 10 µM Compound 12. Immunoblots for (**a-c**) are representative of two independent experiments (n=2). The samples were derived from the same experiment, but different gels for p-OXSR1(S325), another for p-P70S6K (T389), another for P70S6K (Total) and another for Vinculin were processed in parallel in (**a**). The samples were derived from the same experiment, but different gels for p-AKT(S473), another for p-AKT(T308), another for pan-AKT(Total), and another for p-GSK3β (S9) were processed in parallel in (**b**). The samples were derived from the same experiment, but different gels for p-AMPK(T172), another for AMPK(Total), another for p-Beclin-1 (S93), another for Beclin-1 (Total), and another for and Vinculin were processed in parallel in (**c**). Ponceau S staining and Vinculin served as loading controls. (**d**) Immunoblot analysis of the indicated proteins in Cas9-expressing *Wnk1*$^{fl/-}$ MA9 leukaemia cells that express sgRNAs targeting *Pras40* after treatment with DMSO or 10 µM Compound 12 for 1 h. Immunoblots are representative of two independent experiments (n=2). The samples were derived from the same experiment, but different gels for AMPK and p-ULK1(S555), another for p-P70S6K (T389), p-4EBP1(T37/46) and Vinculin, and another for P70S6K (Total) and 4EBP1 (Total) were processed in parallel. Ponceau S staining and Vinculin served as loading controls. (**e**) Immunoblot analysis of the indicated proteins in Cas9-expressing *Wnk1*$^{fl/-}$ MA9 leukaemia cells that express sgRNAs targeting *Prkaa1*(AMPKalpha1) after treatment with DMSO or 10 µM Compound 12 for 1 h. Immunoblots are representative of two independent experiments (n=2). The samples were derived from the same experiment, but different gels for PRAS40 and Vinculin, another for p-P70S6K (T389) and p-4EBP1(T37/46), and another for P70S6K (Total) and 4EBP1 (Total) were processed in parallel. Ponceau S staining and Vinculin served as loading controls. (**f**) Growth curve of *Wnk1*$^{fl/-}$ MA9 leukaemia cells deleting *Nprl2* with three individual sgRNAs treated with/without 500 nM OHT. Ethanol (EtOH) was used as a control. Data are presented as mean ± SD of three biological replicates. (**g**) Immunoblot analysis of the indicated proteins in FKBP12F36V-WNK1 leukemia cells re-stimulated with 2 mM Leu or 4 mM Gln following amino acid starvation (-AA). WNK1 inhibition was achieved by adding dTAG-13 or WNK463 at the start of amino acid starvation. Cells were starved of amino acids for 1 hour, after which Leu or Gln was added, either with or without dTAG-13 or WNK463, for another hour. Blots are representative of two independent experiments (n=2). The samples were derived from the same experiment, but different gels for WNK1 and p-OXSR1(S325), and another for p-P70S6K (T389) and Vinculin were processed in parallel. Vinculin served as a loading control. (**h**) Drop-out growth competition assays showing the relative percentage over time of the sgRNA-positive (GFP positive) Cas9-expressing leukaemia cells that ectopically express the indicated sgRNAs. An sgRNA against an essential gene (*Rps19* for mouse leukaemia cells, and *RPS19* for human AML cells) was used as a positive control, and a non-targeting sgRNA (NCtrl) was used as a negative control. Data shown for each cell line are from one representative experiment (n=1).

Supplementary Fig.9 Uncropped scans of blots shown in Fig.1,Fig.2,Fig4.

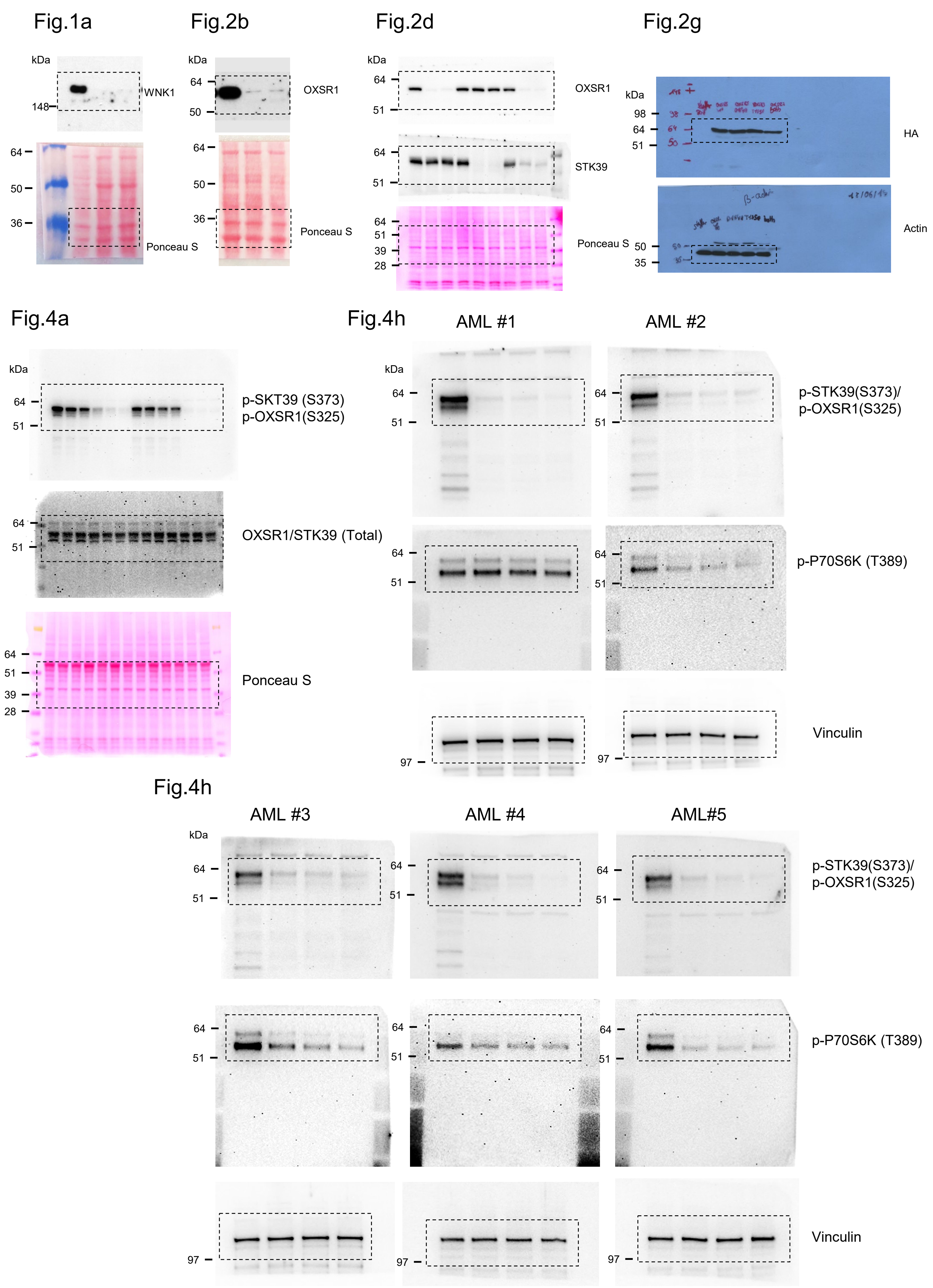

Supplementary Fig.10 Uncropped scans of blots shown in Fig.5d and Fig.5e.

Fig.5d

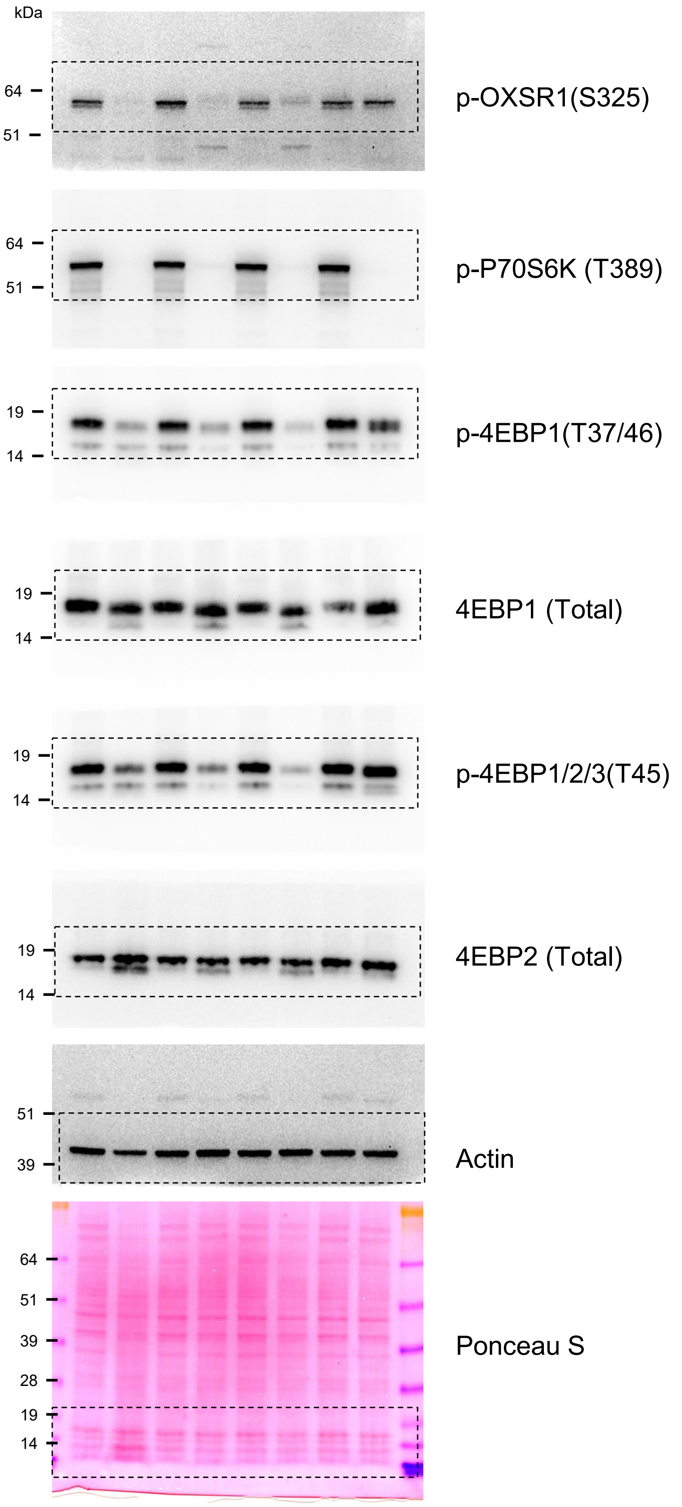

Fig.5e

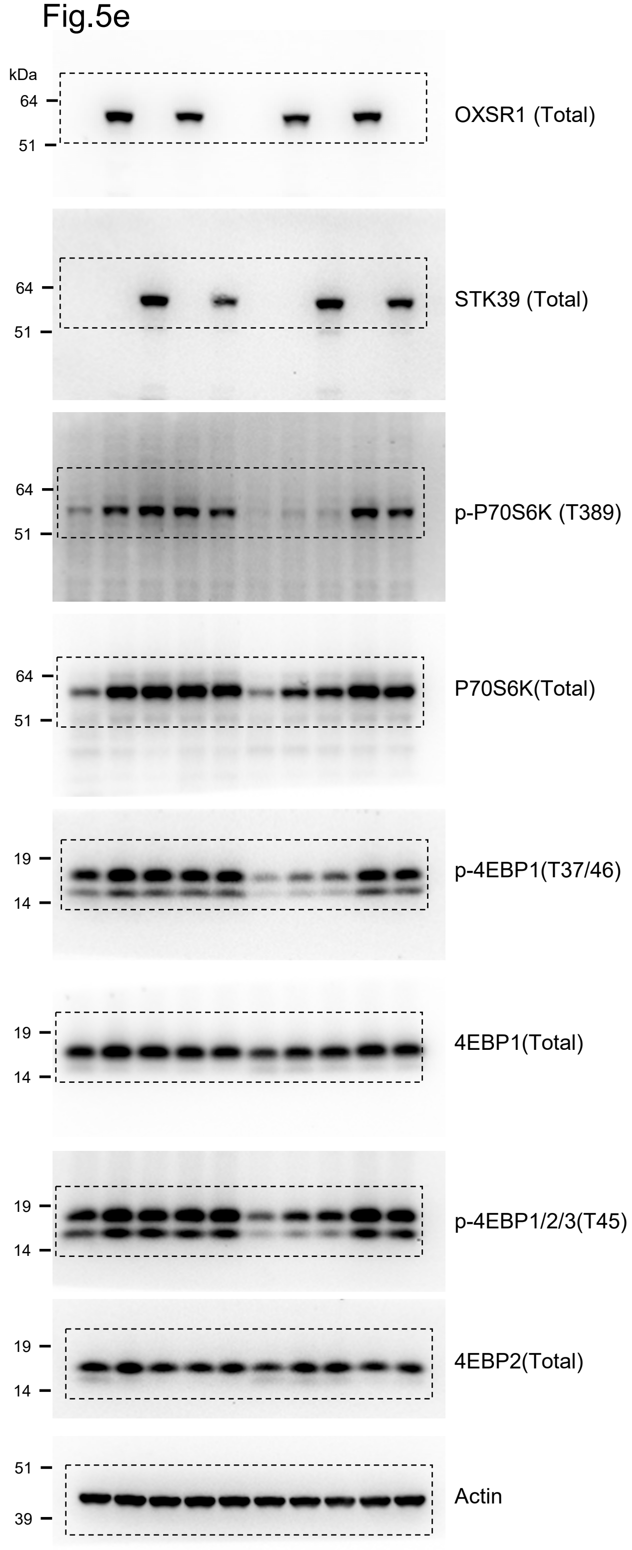

Supplementary Fig.11 Uncropped scans of blots shown in Fig.5f and Fig.6a.

Fig.5f

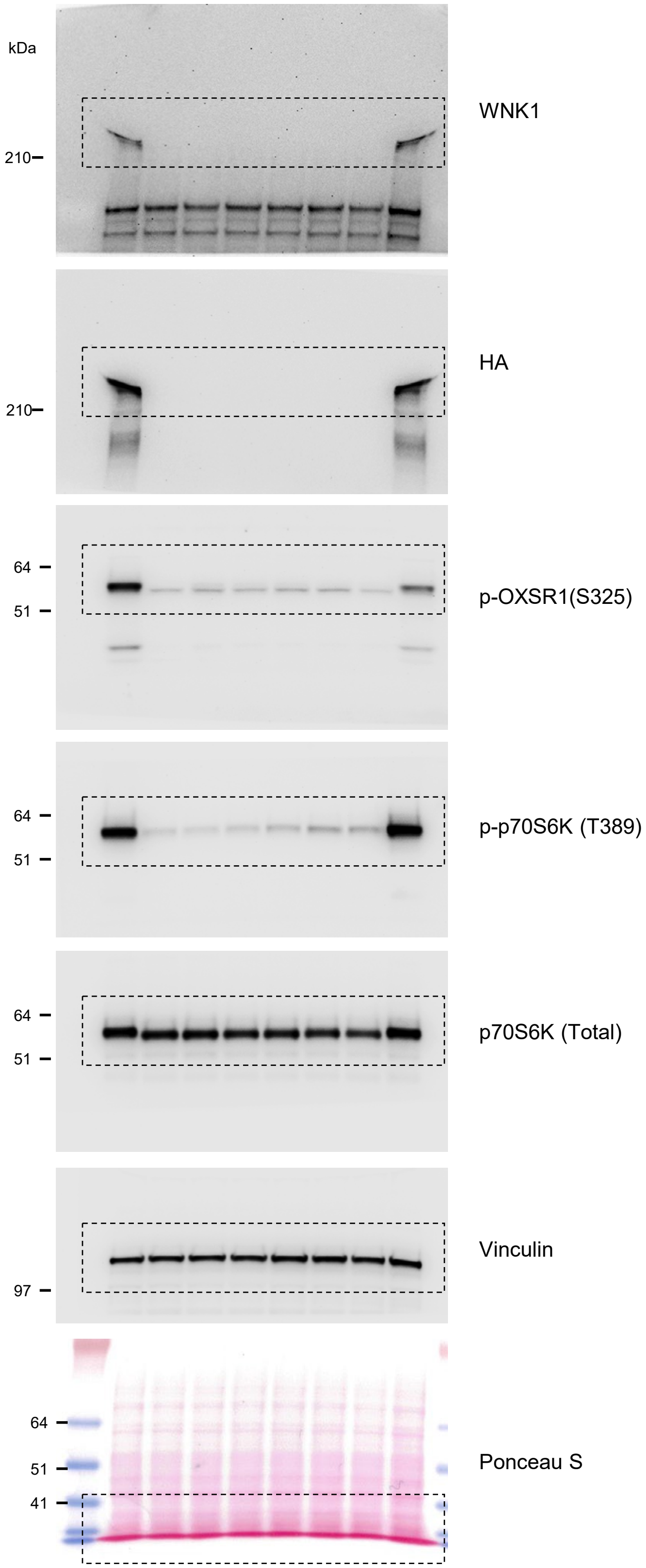

Fig.6a

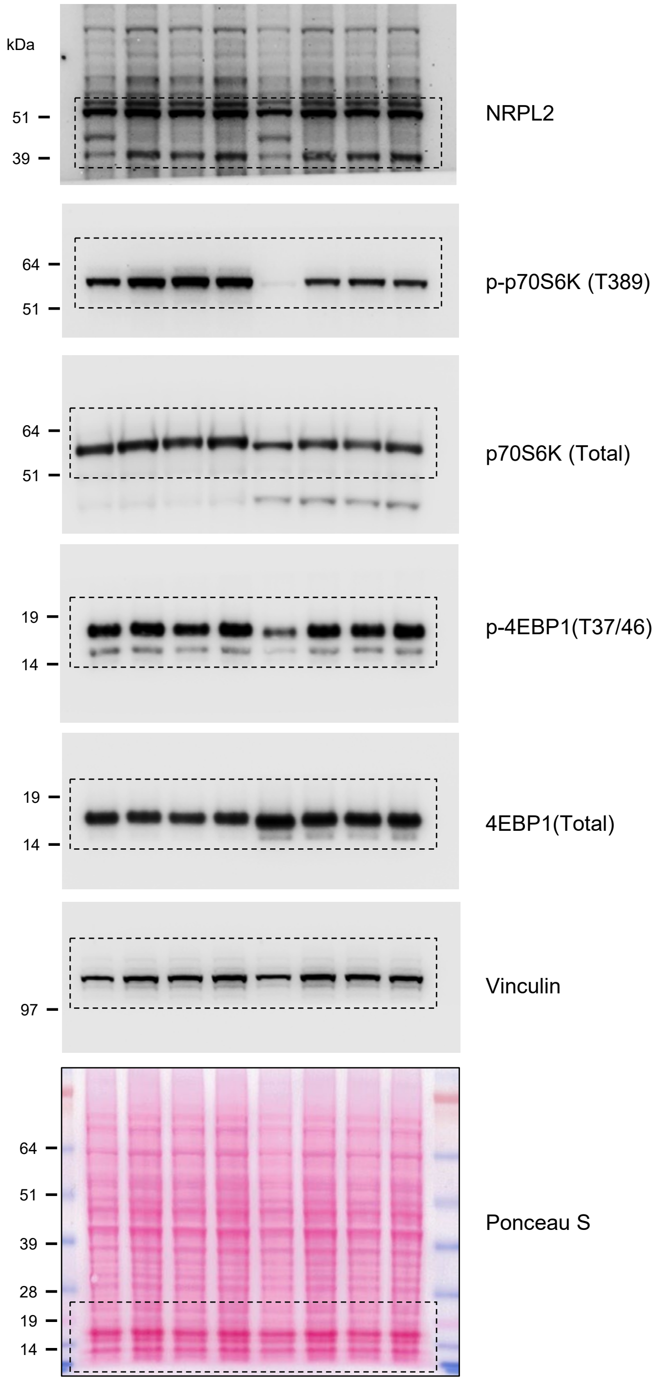

Supplementary Fig.12: Uncropped scans of blots/gels shown in Fig.6f, Fig.6g and Fig.6h.

Fig.6f

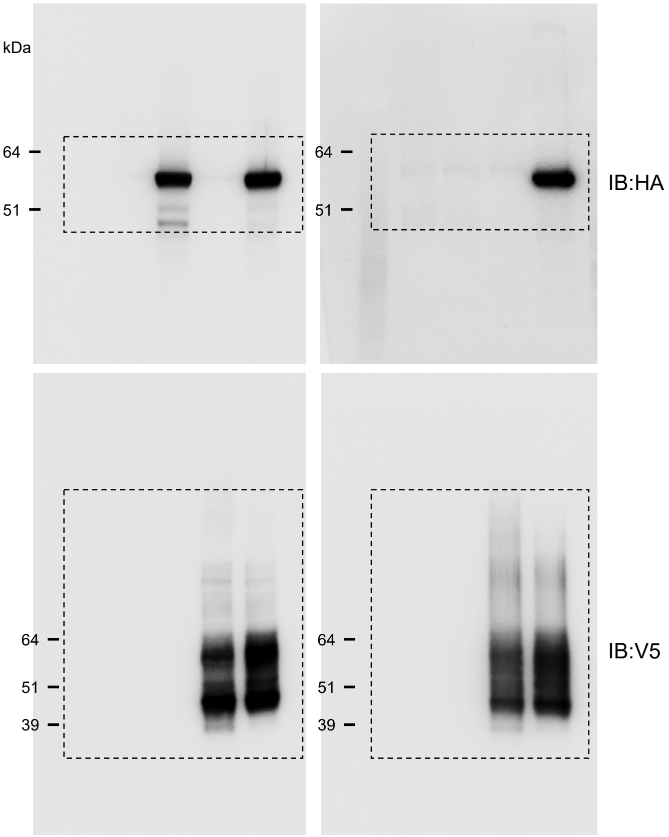

Fig.6g

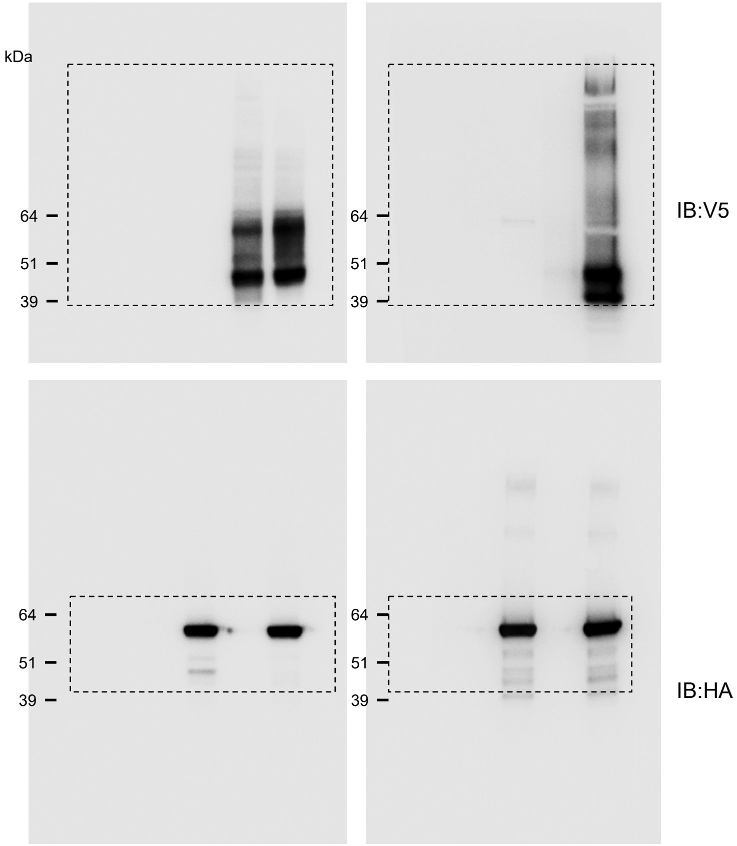

Fig.6h

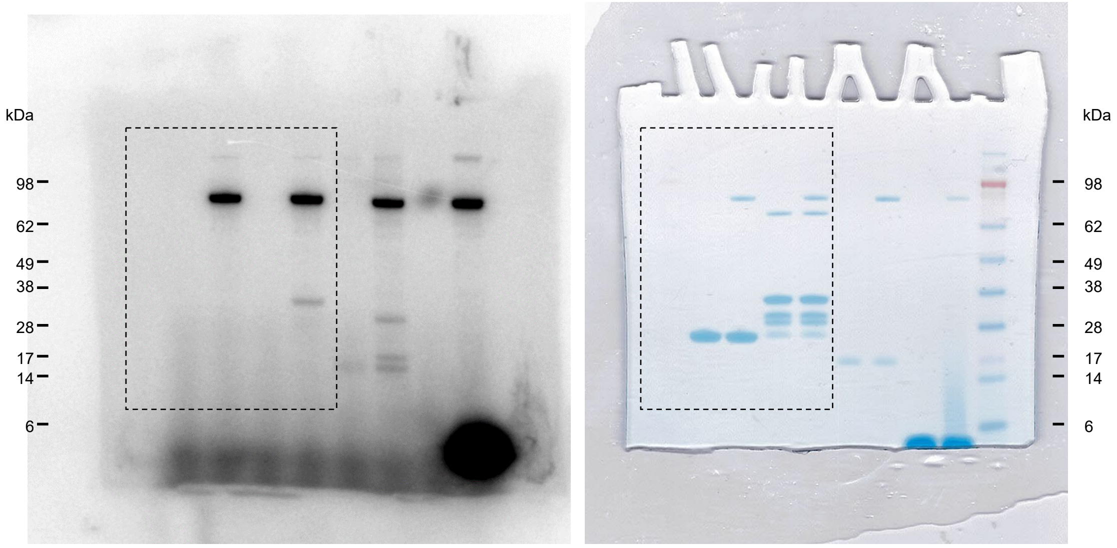

Supplementary Fig.13: Uncropped scans of gels/blots shown in Supplementary Fig.2 and Supplementary Fig.3.

Supplementary Fig.2a

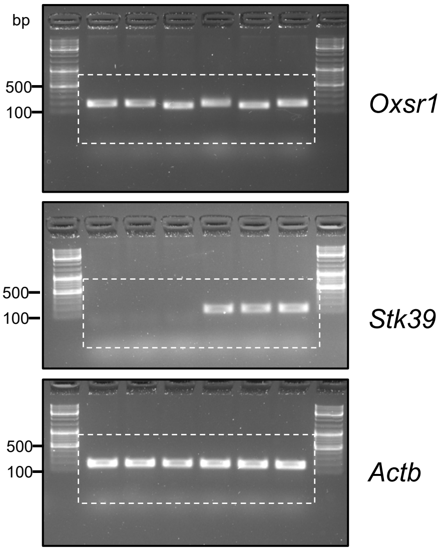

Supplementary Fig.2b

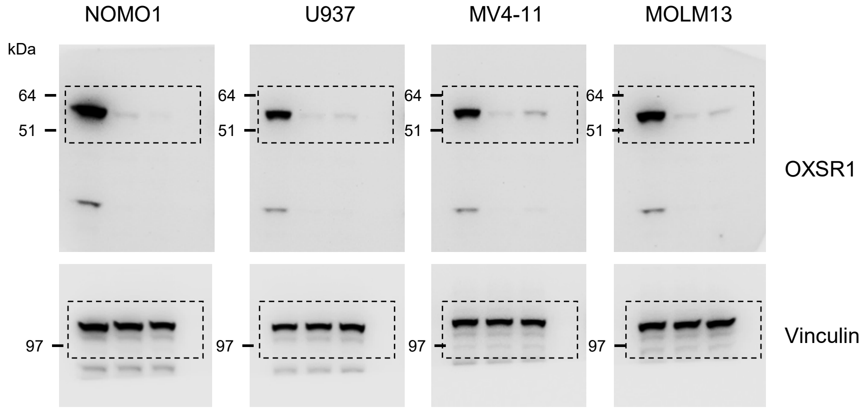

Supplementary Fig.2c

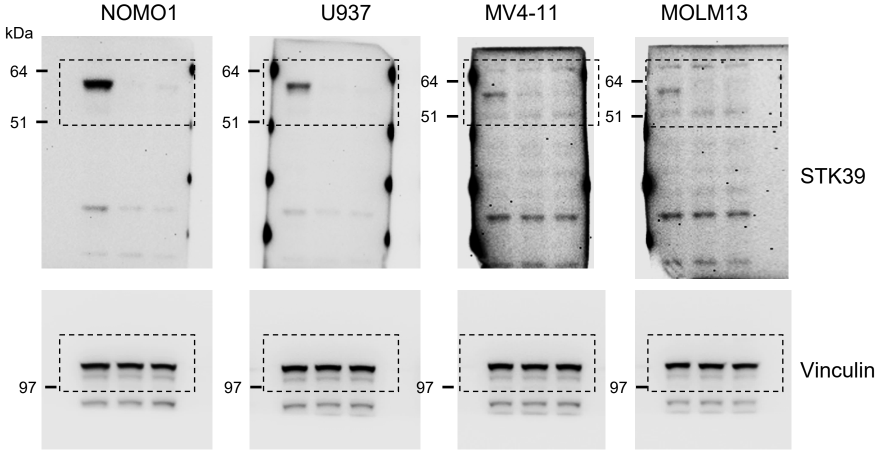

Supplementary Fig.3a

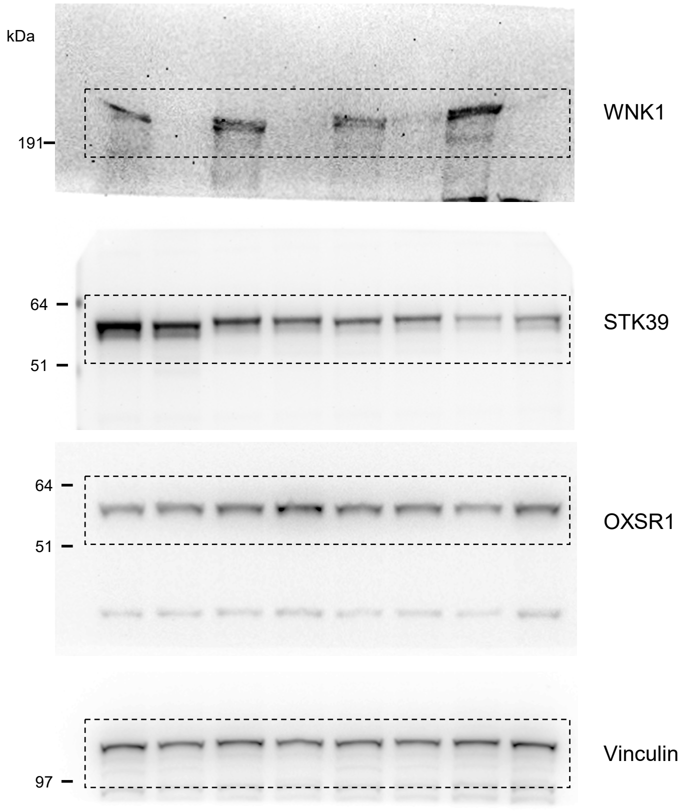

Supplementary Fig.3b

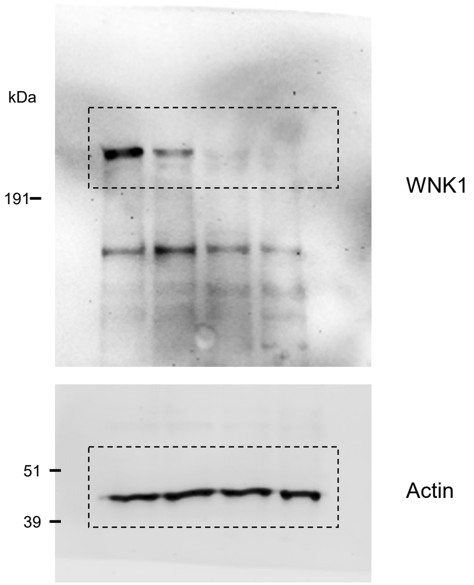

Supplementary Fig.14: Uncropped scans of gels/blots shown in Supplementary Fig.3 and Supplementary Fig.6.

Supplementary Fig.3e

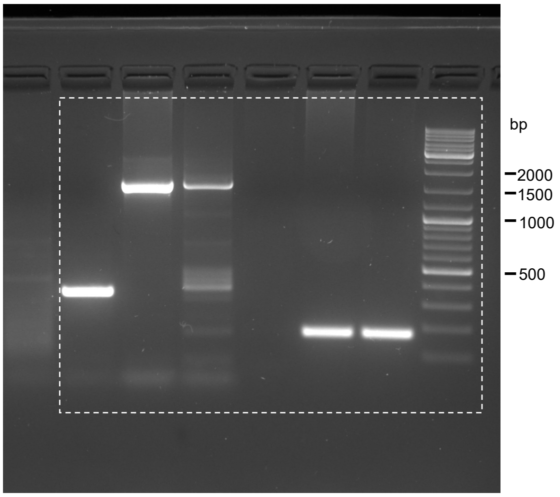

Supplementary Fig.3g

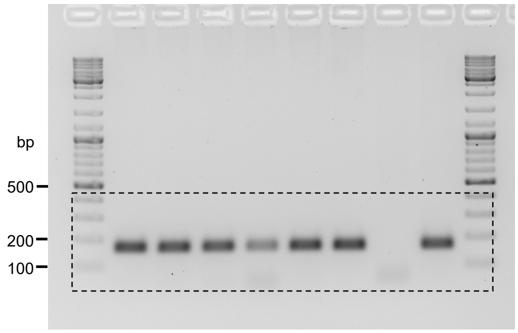

Supplementary Fig.3l

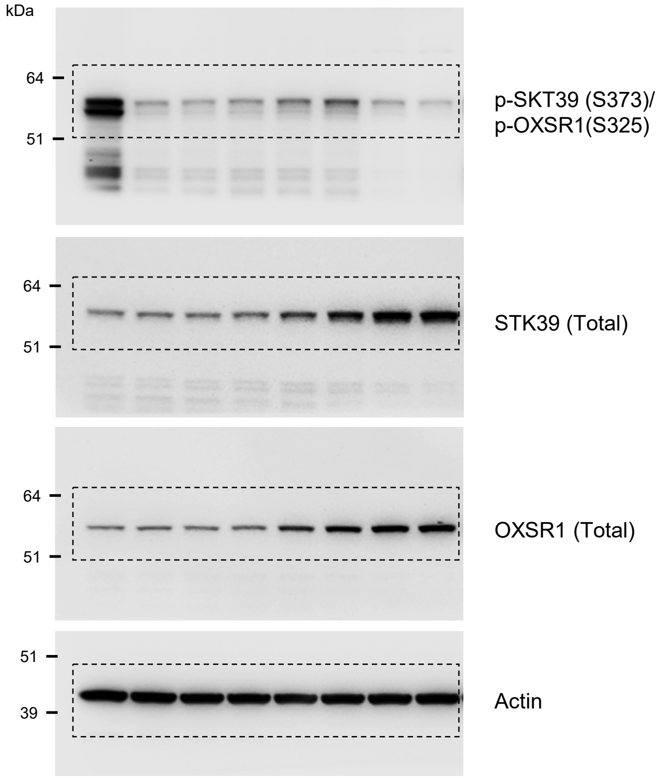

Supplementary Fig.6a

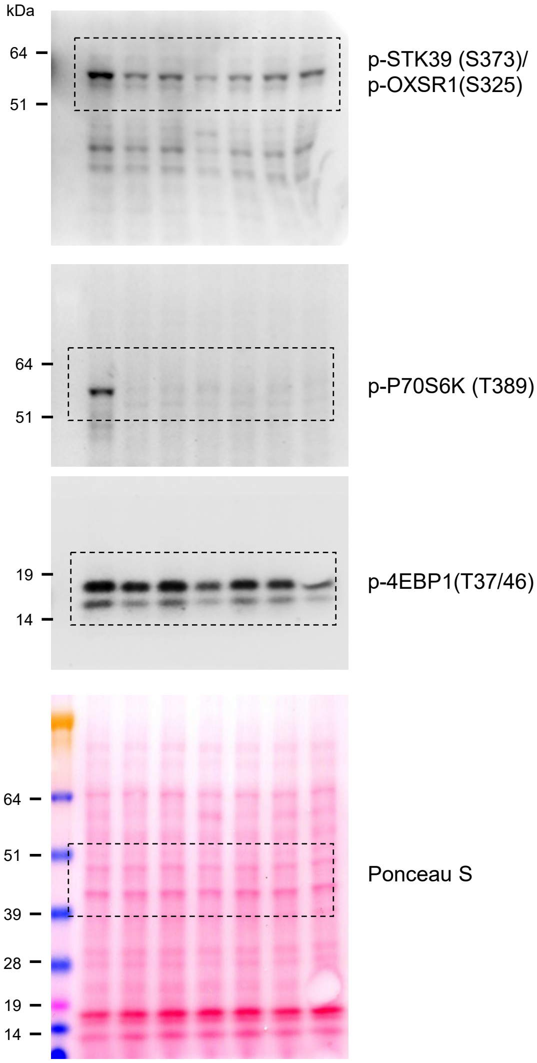

Supplementary Fig.15: Uncropped scans of blots shown in Supplementary Fig.6c,e.

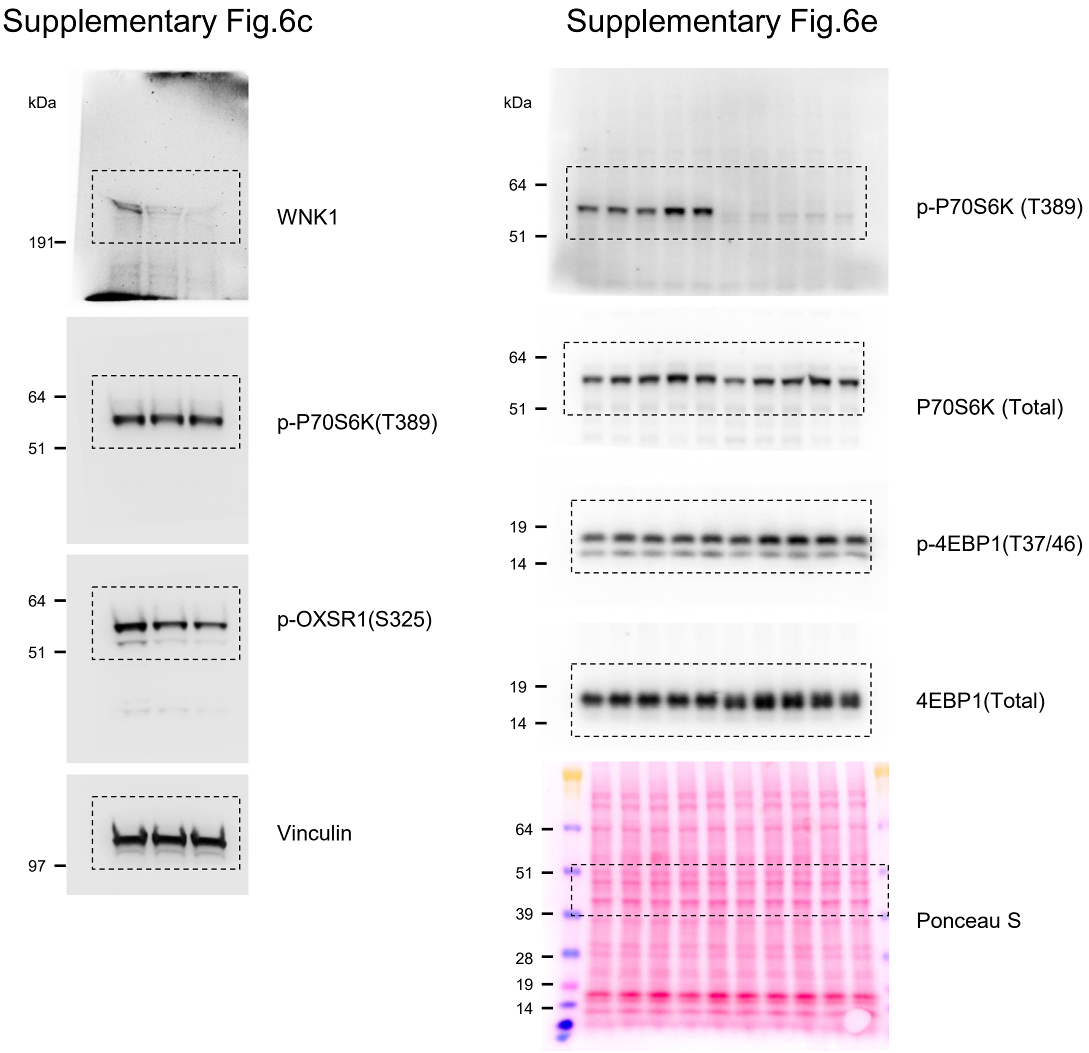

Supplementary Fig.16: Uncropped scans of blots shown in Supplementary Fig.8a-c.

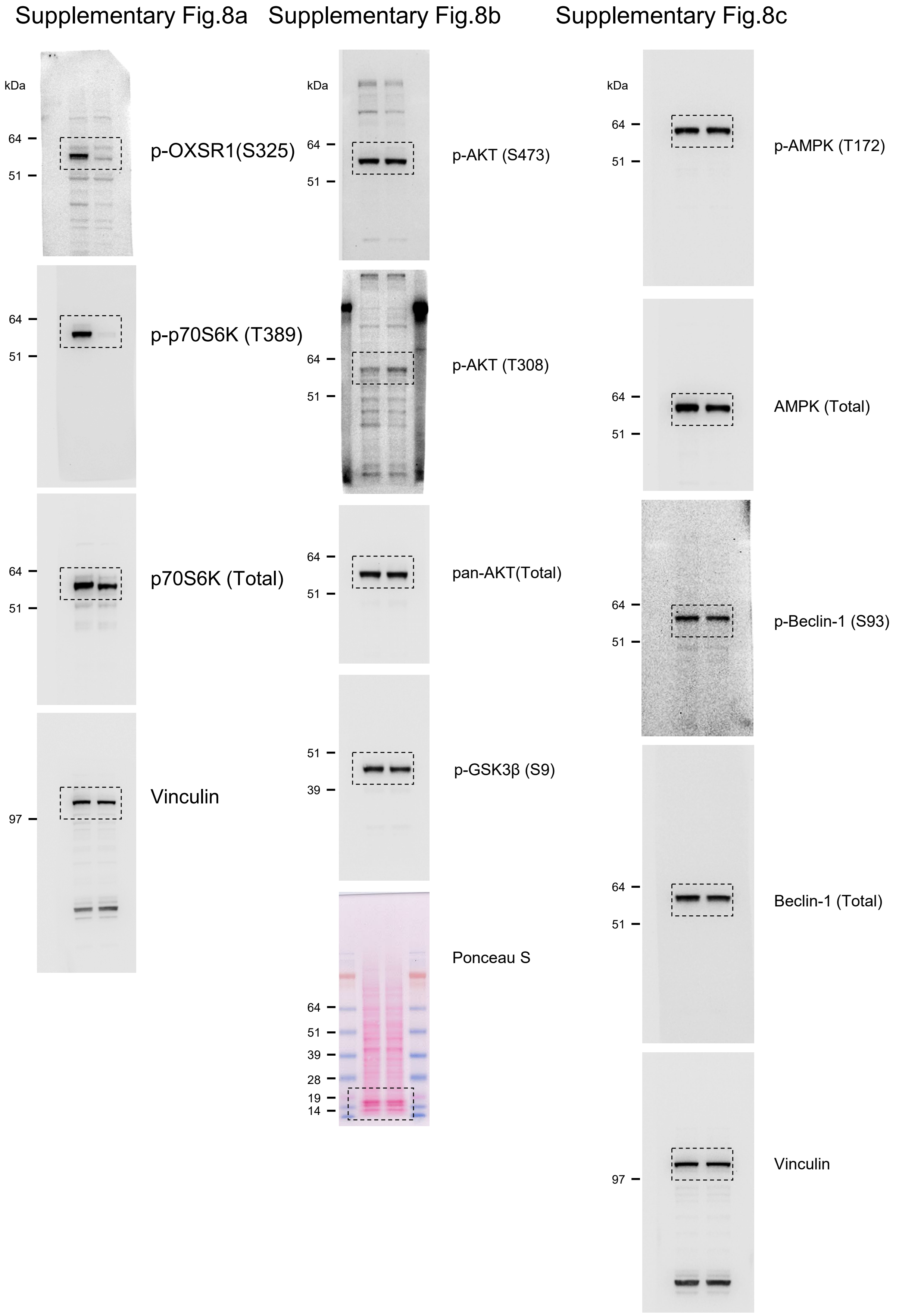

Supplementary Fig.17: Uncropped scans of blots shown in Supplementary Fig.8d,e.

Supplementary Fig.8d

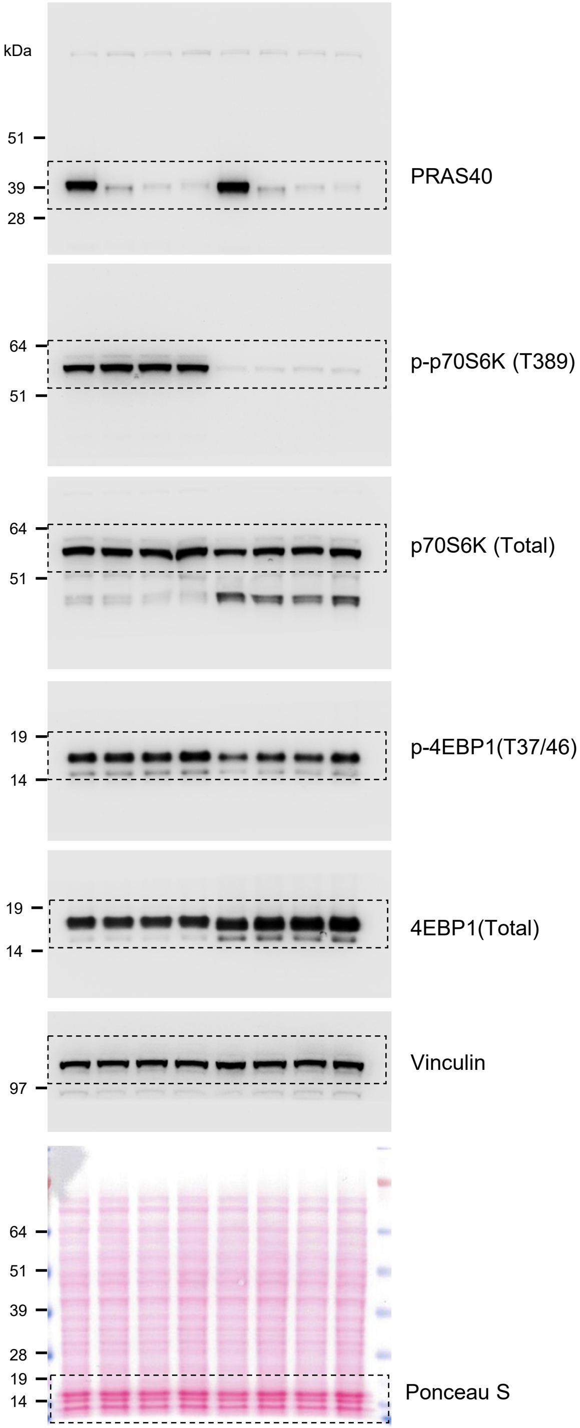

Supplementary Fig.8e

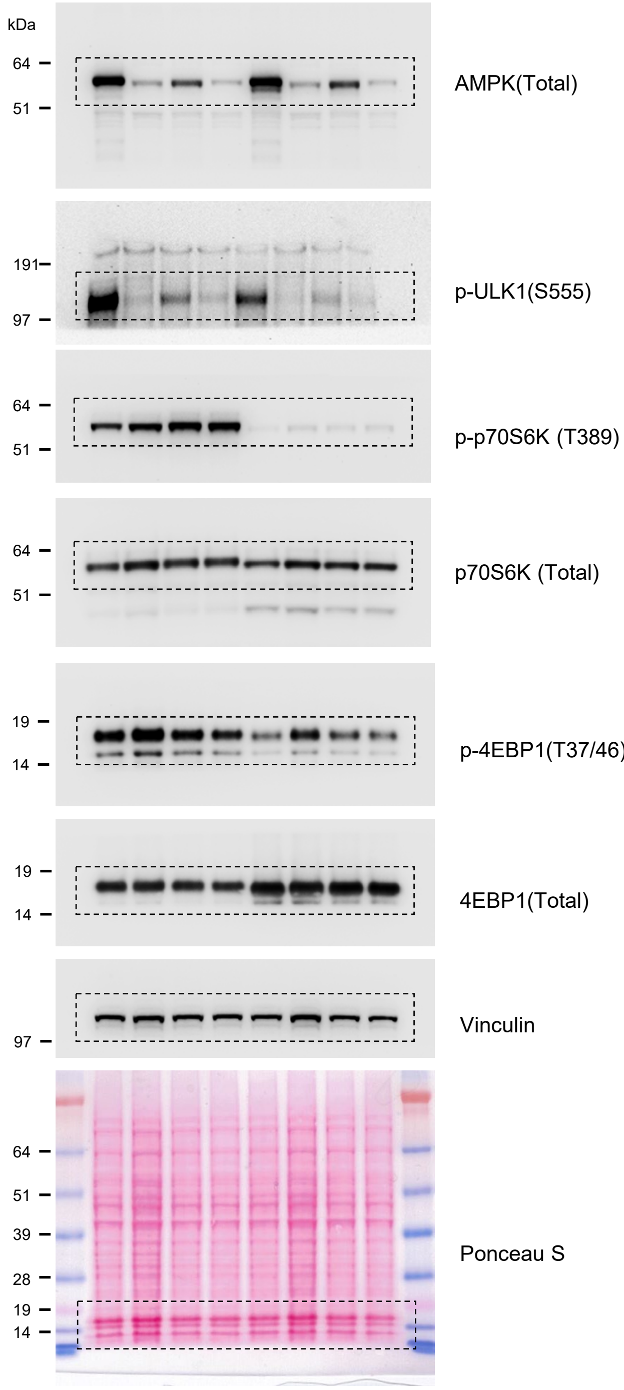

Supplementary Fig.18: Uncropped scans of blots shown in Supplementary Fig.8g.

Supplementary Fig.8g

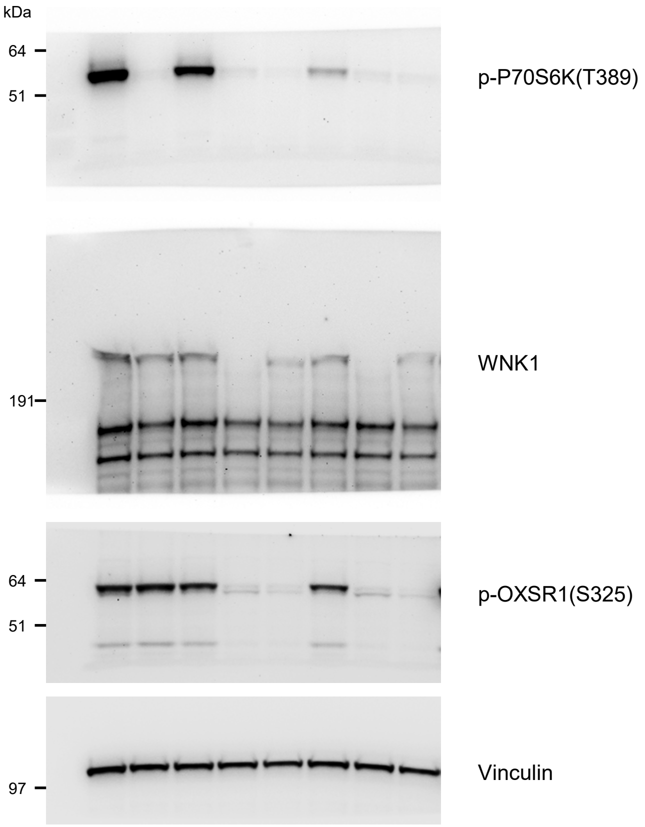

Supplementary Fig.19 Flow cytometry plots showing the gating strategy used to identify OP-Puro–incorporated cells. Related to Fig.5g and Supplementary Fig. 7a-b.

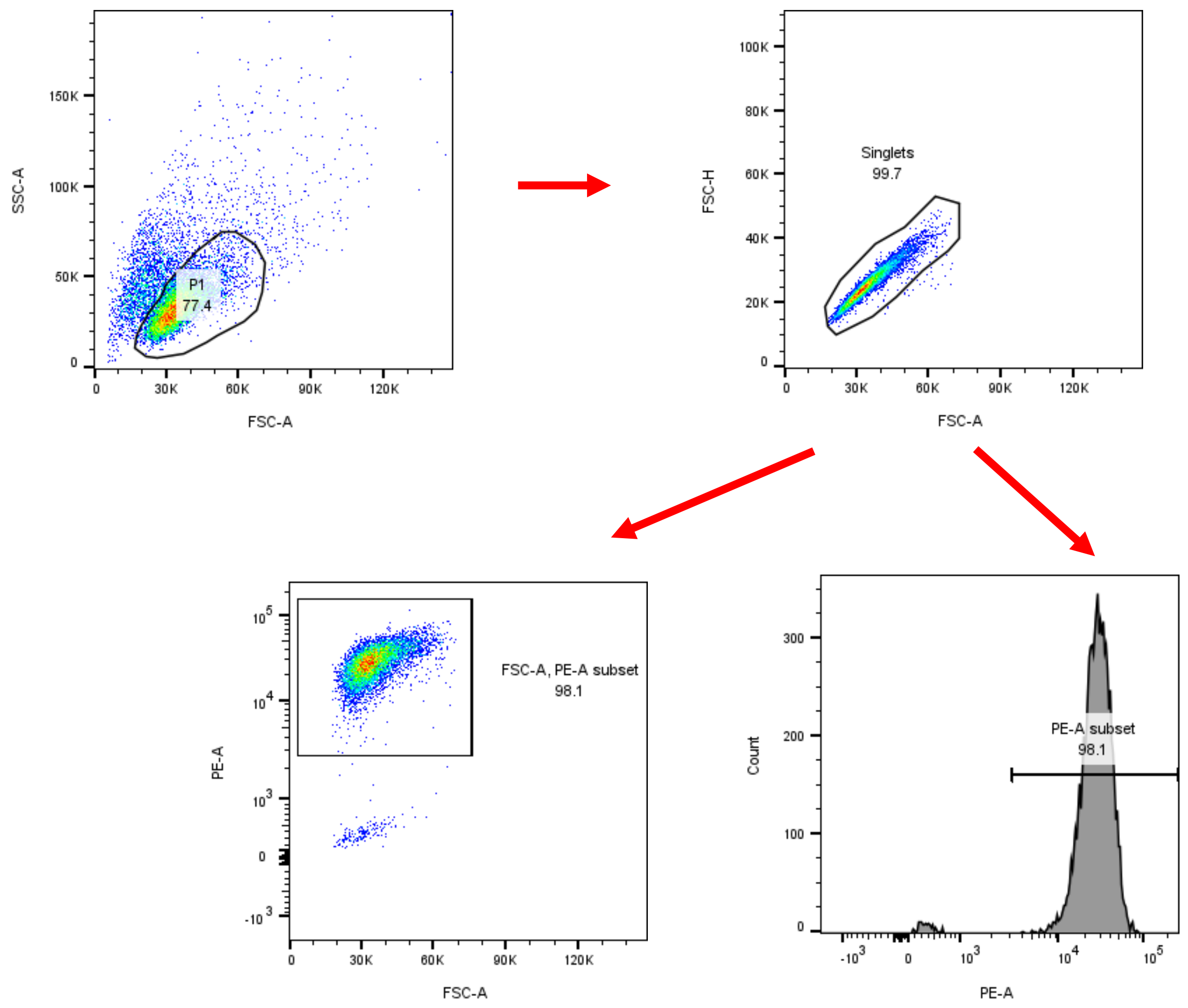

Supplement: Supplementary file 1 — Supplementary information [file 41467_2025_59969_MOESM1_ESM.pdf]
