# Supplementary material for: WNK1 signalling regulates amino acid transport and mTORC1 activity to sustain acute myeloid leukaemia growth
Source: Nat Commun. 2025 May 27;16:4920. doi: 10.1038/s41467-025-59969-8 (PMC12116911; doi:10.1038/s41467-025-59969-8)
Supplement: Supplementary file 2 — Description of additional supplementary information [file 41467_2025_59969_MOESM2_ESM.docx]

Legends for all supplementary data files

Supplementary Data 1. Primary AML samples used in this study. Related to Fig.4h-j and Supplementary Fig. 5a-c.

Supplementary Data 2. Quantitative results for 6,350 unique proteins in *Wnk1^f/-^* MA9 cells treated with either EtOH or OHT, and with either DMSO or Compound 12.

Supplementary Data 3. Quantitative results for 21,725 phosphosites corresponding to 9,471 unique phosphopeptide sequences in *Wnk1^f/-^* MA9 cells treated with either EtOH or OHT, and with either DMSO or Compound 12. The phosphopeptide data was normalised to the corresponding protein data.

Supplementary Data 4. Quantitative metabolomic results for amino acids in *Wnk1^f/-^* MA9 cells treated with either EtOH or OHT, and with either DMSO or Compound 12, using the AbsoluteIDQTM p180 kit.

Supplementary Data 5. Quantitative results for phosphosites of SLC transporters in *Wnk1^f/-^* MA9 cells treated with either EtOH or OHT, and with either DMSO or Compound 12.

Supplementary Data 6. Sequences of sgRNAs and PCR primers used in this study.

Supplementary Data 7. List of antibodies used in this study.
