# Supplementary material for: WNK1 signalling regulates amino acid transport and mTORC1 activity to sustain acute myeloid leukaemia growth
Source: Nat Commun. 2025 May 27;16:4920. doi: 10.1038/s41467-025-59969-8 (PMC12116911; doi:10.1038/s41467-025-59969-8)
Supplement: Supplementary file 10 — Reporting summary [file 41467_2025_59969_MOESM10_ESM.pdf]

nature portfolio

Corresponding author(s): KRISTIAN HELIN

Last updated by author(s): Apr 24, 2025

Reporting Summary

Nature Portfolio wishes to improve the reproducibility of the work that we publish. This form provides structure for consistency and transparency in reporting. For further information on Nature Portfolio policies, see our Editorial Policies and the Editorial Policy Checklist.

Statistics

For all statistical analyses, confirm that the following items are present in the figure legend, table legend, main text, or Methods section.

n/a	Confirmed	
[ ]	[x]	The exact sample size (*n*) for each experimental group/condition, given as a discrete number and unit of measurement
[ ]	[x]	A statement on whether measurements were taken from distinct samples or whether the same sample was measured repeatedly
[ ]	[x]	The statistical test(s) used AND whether they are one- or two-sided<br>*Only common tests should be described solely by name; describe more complex techniques in the Methods section.*
[x]	[ ]	A description of all covariates tested
[ ]	[x]	A description of any assumptions or corrections, such as tests of normality and adjustment for multiple comparisons
[ ]	[x]	A full description of the statistical parameters including central tendency (e.g. means) or other basic estimates (e.g. regression coefficient) AND variation (e.g. standard deviation) or associated estimates of uncertainty (e.g. confidence intervals)
[ ]	[x]	For null hypothesis testing, the test statistic (e.g. *F*, *t*, *r*) with confidence intervals, effect sizes, degrees of freedom and *P* value noted<br>*Give P values as exact values whenever suitable.*
[x]	[ ]	For Bayesian analysis, information on the choice of priors and Markov chain Monte Carlo settings
[x]	[ ]	For hierarchical and complex designs, identification of the appropriate level for tests and full reporting of outcomes
[x]	[ ]	Estimates of effect sizes (e.g. Cohen's *d*, Pearson's *r*), indicating how they were calculated

Our web collection on statistics for biologists contains articles on many of the points above.

Software and code

Policy information about availability of computer code

Data collection	Flow cytometry data was collected using LSRFortessa™ X-20 (BD Biosciences), BD FACSAria III (BD Biosciences), CytoFLEX LX (Beckman) ; Western blot data was collected using ChemiDoc MP Imaging System (Bio-Rad); Autoradiography data was collected using Amersham Typhoon IP Phosphor Imaging Scanner; Sequencing data was collected using Next-Seq550 (Illumina); LC-MS metabolomics data was collected using Waters Acquity H-class UPLC coupled to a Xevo TQ-S triple-quadrupole MS/MS System (Waters); Phosphoproteome data was collected using Orbitrap Fusion Lumos Tribrid Mass Spectrometer (Thermo Fisher); LC-MS data for glutamine uptake was collected using Agilent 1290 Infinity II LC system and Agilent Accurate Mass 6545 Q-TOF instrument (Agilent).
Data analysis	FlowJo (v10) was used to analyze flow cytometry data; DEseq2 was used to analyse CRISPR/Cas9 screen data; GraphPad Prism software(v9) was used to perform statistical analysis. MassLynx™ and TargetLynx™ software (Waters, Manchester, UK) and the MetIDQTM software package (Biocrates Life Sciences AG) were used to performed LC-MS metabolomics data analysis. Proteome Discoverer software (v3.1), R, MSstatsTMT and clusterProfiler were used to analyse phosphoproteome data; Agilent Profinder software (version B.8.0.00 service pack 3) was used to analyse LC-MS data for glutamine uptake.

For manuscripts utilizing custom algorithms or software that are central to the research but not yet described in published literature, software must be made available to editors and reviewers. We strongly encourage code deposition in a community repository (e.g. GitHub). See the Nature Portfolio guidelines for submitting code & software for further information.

Data

Policy information about availability of data

All manuscripts must include a data availability statement. This statement should provide the following information, where applicable:

- Accession codes, unique identifiers, or web links for publicly available datasets
- A description of any restrictions on data availability
- For clinical datasets or third party data, please ensure that the statement adheres to our policy

Proteomics data are available via ProteomeXchange with identifier PXD041902. Source data are provided with this paper

Research involving human participants, their data, or biological material

Policy information about studies with human participants or human data. See also policy information about sex, gender (identity/presentation), and sexual orientation and race, ethnicity and racism.

Reporting on sex and gender	Reporting on sex and gender was summarized in Supplementary Table 1
Reporting on race, ethnicity, or other socially relevant groupings	NA
Population characteristics	Population characteristics were summarized in Supplementary Table 1
Recruitment	N/A
Ethics oversight	Primary human AML samples were obtained from the Barts Cancer Institute Biobank (Referebce number:BCITB-SD-[CID]-1488434). All samples were collected and studied following informed consent and approval by the Barts Cancer Institute Ethical Committees and the BCI Tissue Biobank's scientific sub-committee, in compliance with the Declaration of Helsinki.

Note that full information on the approval of the study protocol must also be provided in the manuscript.

Field-specific reporting

Please select the one below that is the best fit for your research. If you are not sure, read the appropriate sections before making your selection.

[x] Life sciences [ ] Behavioural & social sciences [ ] Ecological, evolutionary & environmental sciences

For a reference copy of the document with all sections, see nature.com/documents/nr-reporting-summary-flat.pdf

Life sciences study design

All studies must disclose on these points even when the disclosure is negative.

Sample size	The number of mice assigned to each treatment arm was selected to provide sufficient statistical power to discern significant differences. For all other experiments, a sample size of at least n=3 was chosen to ensure statistical significance.
Data exclusions	No data exclusions were made in this study.
Replication	The stated number of replicates for each experiment can be found in the figure legends.
Randomization	Mice were randomized into groups, accounting for age and sex factors.
Blinding	The researchers were not blinded to the experiments, and the results were obtained using objective quantitative methods.

Reporting for specific materials, systems and methods

We require information from authors about some types of materials, experimental systems and methods used in many studies. Here, indicate whether each material, system or method listed is relevant to your study. If you are not sure if a list item applies to your research, read the appropriate section before selecting a response.

Materials & experimental systems

n/a	Involved in the study	
	☒	Antibodies
	☒	Eukaryotic cell lines
☒		Palaeontology and archaeology
	☒	Animals and other organisms
☒		Clinical data
☒		Dual use research of concern
☒		Plants

Methods

n/a	Involved in the study	
☒		ChIP-seq
	☒	Flow cytometry
☒		MRI-based neuroimaging

Antibodies

Antibodies used

SPAK, Cell Signaling, 2281s, WB, 1:1000
OSR1, Cell Signaling, 3729s , WB, 1:1000
WNK1, Cell Signaling, 4979s, WB, 1:1000
Vinculin, Sigma , hVIN-1, WB, 1:10000
β-Actin, Abcam , ab6276, WB, 1:10000
HA-tag, Cell Signaling, 3724s, WB, 1:5000
phospho-SPAK (Ser373)/phospho-OSR1 (Ser325), Merck Millipore, 07-2273, WB, 1:1000
Goat Anti-Rabbit IgG -HRP, Vector Laboratories, PI-1000, WB,1:5000
Horse Anti-Mouse IgG -HRP, Vector Laboratories, PI-2000, WB,1:5000
Phospho-Akt (Ser473) , Cell Signaling, 9271S, WB, 1:1000
Phospho-Akt (Thr308), Cell Signaling, 9275S, WB, 1:1000
Akt (pan) (C67E7), Cell Signaling, 4691S, WB, 1:1000
Anti-V5 Agarose Affinity Gel , Sigma, A7345-1ML, IP
V5 Tag , Sigma, V8012-50UG, WB, 1:5000
Phospho-4E-BP1 (Thr37/46), Cell Signaling, 2855S, WB, 1:1000
4E-BP1 (53H11) , Cell Signaling, 9644S, WB, 1:1000
Phospho-p70 S6 Kinase (Thr389), Cell Signaling, 9234S, WB, 1:1000
p70 S6 Kinase (49D7) , Cell Signaling, 2708S, WB, 1:1000
4E-BP2 Antibody, Cell Signaling, 2845S, WB, 1:1000
eIF4EBP1 + eIF4EBP2 + eIF4EBP3 (phospho T45), Abcam, ab68187, WB, 1:1000
Pierce™ Anti-HA Magnetic Beads, Thermo Fisher Scientific, 88836, IP
Phospho-AMPKα, Cell Signaling, 50081S, WB, 1:1000
AMPKα (D5A2) , Cell Signaling, 5831, WB, 1:1000
Phospho-ULK1 (Ser555) (D1H4), Cell Signaling, 5869, WB, 1:1000
ULK1 (D8H5), Cell Signaling, 8054, WB, 1:1000
Beclin-1 (D40C5), Cell Signaling, 3495, WB, 1:1000
Phospho-Beclin-1 (Ser93) (D9A5G), Cell Signaling, 14717, WB, 1:1000
Phospho-GSK-3β (Ser9) (D85E12), Cell Signaling, 5558, WB, 1:1000
NPRL2 (D8K3X), Cell Signaling, 37344, WB, 1:1000
PRAS40 (D23C7), Cell Signaling, 2691T, WB, 1:1000
CD117, Miltenyi Biotech, 130-091-224, MACS
FITC anti-human CD19 Antibody,BIOLEGEND, FACS,1:100
PE/Cyanine7 anti-human CD33 Antibody,BIOLEGEND,FACS,1:100
PE/Cyanine5 anti-human CD3 Antibody,BIOLEGEND,FACS,1:100
APC anti-human CD45 Antibody,BIOLEGEND,FACS,1:100

Validation

All antibodies used in this study were validated and purchased from commercial vendors. They were utilized in accordance with the manufacturer's instructions.

Eukaryotic cell lines

Policy information about cell lines and Sex and Gender in Research

Cell line source(s)

MA9 leukaemia cell lines were generated in house;
THP1, NOMO1, MOLM13, MV4-11, MonoMac6,U937,MOLM16 cell lines were obtained from DSMZ;
BJ-Tert, H226,MM1S cell lines were obtained from ATCC.

Authentication

No cell authentication was performed

Mycoplasma contamination

Cell were routinely checked for Mycoplasma contamination via PCR and tested negative

Commonly misidentified lines (See ICLAC register)	No commonly misidentified cell lines were used.

Animals and other research organisms

Policy information about studies involving animals; ARRIVE guidelines recommended for reporting animal research, and Sex and Gender in Research

Laboratory animals	C57BL/6 and B6.SJL female mice (6–8 weeks old) were used for Wnk1fl/+, Wnk1fl/−, Wnk1fl/D368A MA9 leukaemia cells bone marrow transplantation. For human primary AML xenotransplantation, eight- to ten-week-old NOD.Cg-KitW-41JTyr+Prkdcscid Il2rgtm1Wjl/ThomJ (NBSGW) mice were used.
Wild animals	This study did not include the use of wild animals.
Reporting on sex	Sex was not taken into consideration in the current study.
Field-collected samples	No field-collected samples were used in the current study.
Ethics oversight	All mouse experiments conducted in Denmark were approved by the Danish Animal Ethics Committee under license number 2017-15-0201-01176. All mouse experiments carried out in the UK were approved by the Animals in Science Regulation Unit (license number: PP5781054).

Note that full information on the approval of the study protocol must also be provided in the manuscript.

Flow Cytometry

Plots

Confirm that:

- [x] The axis labels state the marker and fluorochrome used (e.g. CD4-FITC).
- [x] The axis scales are clearly visible. Include numbers along axes only for bottom left plot of group (a 'group' is an analysis of identical markers).
- [x] All plots are contour plots with outliers or pseudocolor plots.
- [x] A numerical value for number of cells or percentage (with statistics) is provided.

Methodology

Sample preparation	Cultured cell lines
Instrument	LSRFortessa™ X-20 (BD Biosciences), BD FACSAria III (BD Biosciences), CytoFLEX LX (Beckman)
Software	FlowJo (v10)
Cell population abundance	N/A
Gating strategy	FSC-A and FSC-H was used to identify single cells; Cells were separated by viability using DAPI exclusion and AnnexinV for apoptosis assay; Cells were separated by GFP or RFP for growth competition assays.

- [x] Tick this box to confirm that a figure exemplifying the gating strategy is provided in the Supplementary Information.
